# Supplementary material for: Melatonin, temazepam and placebo in hospitalised older patients with sleeping problems (MATCH): a study protocol of randomised controlled trial
Source: BMJ Open. 2019 May 22;9(5):e025514. doi: 10.1136/bmjopen-2018-025514 (PMC6537975; doi:10.1136/bmjopen-2018-025514)
Supplement: Author's manuscript [file bmjopen-2018-025514.draft_revisions.pdf]

BMJ Open is committed to open peer review. As part of this commitment we make the peer review history of every article we publish publicly available.

When an article is published we post the peer reviewers' comments and the authors' responses online. We also post the versions of the paper that were used during peer review. These are the versions that the peer review comments apply to.

The versions of the paper that follow are the versions that were submitted during the peer review process. They are not the versions of record or the final published versions. They should not be cited or distributed as the published version of this manuscript.

BMJ Open is an open access journal and the full, final, typeset and author-corrected version of record of the manuscript is available on our site with no access controls, subscription charges or pay-per-view fees (http://bmjopen.bmj.com).

If you have any questions on BMJ Open's open peer review process please email info.bmjopen@bmj.com

BMJ Open

MelAtonin, Temazepam and plaCebo in Hospitalised older patients with sleeping problems (MATCH): a study protocol of randomised controlled trial

Journal:	*BMJ Open*
Manuscript ID	bmjopen-2018-025514
Article Type:	Protocol
Date Submitted by the Author:	18-Jul-2018
Complete List of Authors:	Stenveld, Fiona; University of Groningen, University Medical Centre Groningen, Depatment of Geriatric Medicine<br>Bosman, Sjanne; University of Groningen, University Medical Centre Groningen, Department of Geriatric Medicine; Medical Centre Leeuwarden, Department of Geriatric Medicine<br>van Munster, Barbara; University of Groningen, University Medical Centre Groningen, Department of Geriatric Medicine; Gelre Hospitals, Department of Geriatrics<br>Beishuizen, Sara; Academic Medical Centre, Department of Internal Medicine, Geriatrics Section<br>Hempenius, Liesbeth; Medical Centre Leeuwarden, Department of Geriatric Medicine<br>van der Velde, Nathalie; Academic Medical Centre, Department of Internal Medicine, Geriatrics Section, Amsterdam Public Health<br>Smidt, Nynke; University of Groningen, University Medical Centre Groningen, Department of Geriatric Medicine; University of Groningen, University Medical Centre, Department of Epidemiology<br>De Roolj, Sophia; University of Groningen, University Medical Centre Groningen, Department of Geriatric Medicine; Academic Medical Centre, Department of Internal Medicine, Geriatrics Section, Amsterdam Public Health
Keywords:	hospitalisation, aged, melatonin, benzodiazepines, sleep, randomised controlled trail

SCHOLARONE™
Manuscripts

Version 3, date 06-07-2018

MelAtonin, Temazepam and plaCebo in Hospitalised older patients with sleeping problems (MATCH): a study protocol of randomised controlled trial.

Fiona Stenveld(1)*, Sjanne Bosman*(1,2), Barbara C van Munster (1,3), Sara J.E. Beishuizen (4), Liesbeth Hempenius(2), Nathalie van der Velde (5), Nynke Smidt (1,6), Sophia E de Rooij (1,5)

Author affiliation:

1. University of Groningen, University Medical Centre Groningen, Department of Geriatric Medicine, Groningen, The Netherlands
2. Department of Geriatric Medicine, Medical Centre Leeuwarden, Leeuwarden, The Netherlands.
3. Department of Geriatrics, Gelre Hospitals, Apeldoorn, The Netherlands.
4. Department of Geriatrics, MC Slotervaart, Amsterdam
5. Department of Internal Medicine, Geriatrics Section, Academic Medical Centre, Amsterdam Public Health, Amsterdam, The Netherlands.
6. University of Groningen, University Medical Centre, Department of Epidemiology, Groningen, The Netherlands.

Correspondence public queries: f.stenveld@umcg.nl , University Centre of Geriatric Medicine, University Medical Centre Groningen. Hanzeplein 1, PO box 30 001, 9700 RB Groningen

Correspondence scientific queries: Principal investigator: Professor Sophia E.de Rooij, s.e.j.a.de.rooij@umcg.nl telephone number 050-3613921, Hanzeplein 1, Postbus 30 001, 9700 RB Groningen. University of Groningen, University Medical Centre Groningen, University Centre for Geriatric Medicine, Groningen, The Netherlands.

Word count:2631 (excluding title page, abstract, references, figures and tables, acknowledgments)

For peer review only

ABSTRACT

Introduction: Hospitalised older patients frequently suffer from inadequate sleep, which can lead to patient distress and delayed recovery from acute illness or surgical procedure. Currently, no evidence-based treatments exist for sleeping problems in hospitalised older patients. Benzodiazepines, such as temazepam, are regularly prescribed by physicians, although they have serious side effects; for older patients in particular. Melatonin is proposed as a safe alternative for sleeping problems in hospitalised older patients, but the efficacy of melatonin is unclear in this population. Therefore, the aim of this study is to investigate the effects of melatonin and temazepam compared to placebo on sleep quality among hospitalised older patients with sleeping problems.
Methods and analysis: This study is a multicentre, randomised, placebo controlled trial. A total of 663 patients will be randomised in a 1:1:1 fashion to receive either melatonin (n=221), temazepam (n=221) or placebo (n=221). The study population consists of hospitalised patients aged 60 years and older, with new or aggravated sleeping problems for which an intervention is needed. The primary outcome is sleep quality measured with the Leeds Sleep Evaluation Questionnaire (LSEQ). Secondary outcomes include sleep parameters measured with actigraphy and medication-related adverse effects.
Ethics and dissemination: This study was approved by the Medical Ethics Committee of the Academic Medical Centre Amsterdam, (No 2015_302). Study findings will be disseminated through presentations at professional and scientific conferences and publications in peer-reviewed journals.
Trial registration number: NTR6908

Keywords: hospitalisation; aged; melatonin; benzodiazepines; sleep; insomnia; randomised Controlled Trial;

Strengths and limitations of this study

- The results of this study will provide knowledge relevant for clinical practice and fill gaps in current guidelines concerning sleep problems in hospitalised older patients.
- To optimize the generalisability of the findings, this is a multicentre study with participants from different wards and minimal exclusions criteria.
- This study investigates, next to the effects, also the adverse events of melatonin and temazepam compared to placebo.
- Patients with cognitive impairment are excluded due to ethical considerations, even though these vulnerable patients may benefit the most from the study outcomes.

INTRODUCTION

Background

Hospitalised older patients frequently experience sleeping problems due to medical -, psychological- or environmental disturbances. The prevalence of sleep problems amongst older patients in medical and surgical wards varies between 30 and 65%.[1–3] Apart from the feeling of tiredness, insufficient sleep has substantial consequences for hospitalised patients. This includes delayed recovery from

For peer review only

acute illness or surgical procedures because of its immunosuppressive effect,[4,5] risk of delirium and falls[6–9] and disturbances in metabolic and endocrine functioning.[10,11]

Thus, adequate treatment of sleeping problems is necessary. Today, it is still unclear how sleeping problems should be treated in the hospital. Non-pharmacological interventions are advised as first choice therapy in hospitalised patients,[12–14] but there is limited evidence to support the effectiveness of non-pharmacological interventions.[15] With regard to pharmacological interventions, there is insufficient evidence to support its effectiveness for improving quality or quantity of sleep in hospitalised patients suffering from poor sleep.[16]

Currently, up to 29% of older hospitalised patients with sleeping problems receive benzodiazepines,[17,18] despite a recent meta-analysis of risks and benefits for short-term treatment of insomnia, which stated that benzodiazepines have little effect and may not outweigh the risks of adverse events, especially in a high risk older population.[19] Older patients have a higher risk of adverse drug events from benzodiazepines due to altered pharmacokinetics and pharmacodynamics. Common adverse drug events include in-hospital falls, delirium, aspiration, and respiratory depression.[20,21] Furthermore, use of benzodiazepine during hospitalisation can lead to prolonged use at home, which might promote chronic use and substance-dependency.[22]

The neuroendocrine hormone melatonin has been proposed as a safer alternative to improve sleep quality because of its limited side effects.[23–26] Melatonin, produced by the pineal gland at night, is well known to influence circadian rhythm and sleep.[27] Two randomised control trials (RCT's) investigated the effectiveness of melatonin compared to placebo in older community dwelling persons with chronic complaints of insomnia and found that melatonin improved sleep quality significantly.[28,29] However, studies on the effectiveness of melatonin for sleeping problems in hospitalised older patients are scarce. Three RCT's have been performed in adult patients on the intensive care unit (ICU),[30–32] showing inconsistent results; one out of three reported a positive effect of melatonin.[31] All three studies were underpowered and none specifically assessed older patients with predefined sleep related problems.

Only one small RCT investigated the effectiveness of melatonin on a general medical ward in adults, which resulted in beneficial effects for melatonin compared to placebo on the sleep onset latency and patient satisfaction.[33]

The overall aim of this study is to establish an evidence-based pharmacological treatment for hospitalised older patients with sleeping problems. In this study, we will investigate the effects of melatonin, temazepam and placebo on sleep quality among hospitalised older patients with sleeping problems.

For peer review only - http://bmjopen.bmj.com/site/about/guidelines.xhtml

Objectives

The primary objective is to investigate the effects of melatonin, temazepam and placebo among hospitalised older patients on self-reported sleep quality.

The secondary objectives are to investigate the effects of melatonin, temazepam and placebo on:

1. Other self-reported sleep parameters: getting to sleep, awakening from sleep, behaviour following wakefulness.

2. Objective sleep parameters: reduction in sleep onset latency, sleep efficiency, number and duration of wake bouts, time awake after sleep onset.

3. Adverse events related to study medication: Incidence of delirium, cognition, number of falls, complications, length of hospital stay in days.

4. Time needed to reach improvement in self-reported sleep quality

5. Proportion of good nights of sleep during study period.

METHODS

Trial design

This study is a multicentre, randomised, placebo-controlled trial. Figure 1 shows the study design of the MATCH-study. This study will be conducted in two university teaching hospitals and in two teaching hospitals in the Netherlands: the Academic Medical Centre (AMC) Amsterdam, the University Medical Centre Groningen (UMCG), Gelre Hospitals Apeldoorn, and the Medical Centre Leeuwarden (MCL). The design of this trial protocol has followed the recommendations of the SPIRIT 2013 statement.[34]

Eligibility criteria

Eligible participants must have the following characteristics:

1. 60 years or older.
2. Admitted to the hospital for a medical or surgical reason.
3. Experiencing new onset or aggravated sleep problems during hospital admission, for which an intervention is needed.
4. Ability to fill out a sleep questionnaire.

People with the following characteristics are not eligible:

1. Inability to speak, understand or write Dutch.
2. Lack of decision making capacity.
3. Previously diagnosed dementia.

For peer review only

For peer review only - http://bmjopen.bmj.com/site/about/guidelines.xhtml

4. Transferred from another hospital to one of the study centres, with insufficient information on previous use of sleep medication.
5. Expected stay in hospital of <48 hours.
6. Concurrent benzodiazepine or melatonin use.
7. Alcohol consumption >13 units/week for women and >20 units/week for men.
8. Drug interactions with melatonin or contraindications for benzodiazepine use.

First selection of eligible patients will be done by the attending physician, if eligible the physician will inform the research team. Final selection will be done by a member of the research team, who also will recruit eligible participants and include them after written informed consent for participation has been obtained.

Randomisation and blinding

After patients have given informed consent, participants will be allocated randomly to either temazepam, melatonin or placebo capsules (ratio is 1:1:1). Randomisation is stratified per centre and department, the strata include the 4 centres (AMC, Gelre hospitals Apeldoorn, MCL and UMCG) and departments (surgical versus medical). A stratified block randomisation is used and generated by the program CASTOR Electronic Data Capture (Castor Electronic Data Capture, Ciwit BV, Amsterdam, the Netherlands, 2017). The researchers are not informed about the block sizes.

All medication used in the study will appear identical to ensure true blinding of the patient, attending physician, nurse and investigators. The study medication is manufactured by Tiofarma, Oud-Beijerland, the Netherlands and will be distributed to the study sites' pharmacies. Study medication must be kept intact at all times; the capsules cannot be broken. All study medication is packed in Single Unit Supply packages, blinded and dispensed by site trial pharmacists. The dispensing pharmacist will record the patient, patient ID and assigned medication by the randomisation process. Storage, delivery, dispensing and destruction of medication will adhere to ICH-GCP regulations.[35]

At the end of the study, when all data is collected, the data manager will lock the database. The information of the study arm (melatonin, temazepam and placebo) will be masked by the data manager, after which analysis can be performed in a blinded way by the researchers. Randomisation method will be broken when the study team has declared the data analysis finished. In case of emergencies or urgent situations unblinding of an individual participant will be done according to the unblinding protocol.

Intervention

After randomisation, all participants receive the allocated medication once daily ante noctem. Study medication will be administered orally starting on the day of study enrolment.

For peer review only - http://bmjopen.bmj.com/site/about/guidelines.xhtml

Melatonin 1 mg and temazepam 10 mg will be used as study medication. The dosage of 1 mg melatonin is chosen based on a recent systematic review, to best mimic the normal physiological circadian rhythm of melatonin by avoiding prolonged supra-physiological blood levels in case of higher dosages.[36] The dosage of 10mg temazepam is the recommend dose for older patients.[37] Study medication will be continued to either a maximum of ten days, or 12 hours before hospital discharge, or after three consecutive nights of good sleep, or until participant decides to withdraw from the study. In case sleep problems do not resolve with study medication, the dosage can be doubled. This decision will be left to the patient's attending physician. Attending physicians are instructed only to consider a double dose when the patient has slept badly for two nights in a row, if there are no other environmental or treatable physical factors and they should take possible adverse effects of study medication into account. Double doses of study medication will be registered in the case report form and in the medical record.

Study medication will be discontinued if a condition occurs that needs treatment with benzodiazepines (e.g. anxiety), if events occur that imply a higher risk of adverse events (e.g. respiratory insufficiency or starting chinolones) and in case of persisting sleeping problems. Persisting is defined as sleeping problems for three consecutive nights after study medication has been doubled.

After study medication is discontinued, either due to a reason listed above or due to end of study period, treatment will be according to the hospital's protocol and under the responsibility of the attending physician. Patients remain in the study and follow the study protocol, except for using study medication.

Outcome measures and assessment points

Table 1 provides an overview of the instruments used in this study and the timing of measurements. The primary outcome of the study is change in sleep quality during the treatment period. Sleep quality will be assessed with the Leeds Sleep Evaluation Questionnaire (LSEQ).[38,39] The secondary outcomes, change in self-reported getting to sleep, awakening from sleep and behaviour following wakefulness during treatment period will be assessed with the other subscales of the LSEQ. To assess the objective sleep parameters patients will wear a MotionWatch 8 and to assess the number of good night sleep a numeric rating scale for quality of sleep will be used. On the numeric rating scale ≤ 4 is considered a bad night's sleep, 5-6 is neither a good or bad night's sleep and ≥ 7 is a good night's sleep.[40] Time needed to reach improvement in self-reported sleep quality will be based on the LSEQ as well. Improvement of sleep quality is defined as the first day that an improvement of 10mm is reached compared to baseline. Next to measurement instruments, demographic data, medication use and blood tests 24 hours prior to study inclusion or 24 hours after study inclusion, will also be

For peer review only - http://bmjopen.bmj.com/site/about/guidelines.xhtml

Measurement	Description and instrument	Time points		
		baseline	daily	discharge
**Sleep quality**	*Leeds Sleep Evaluation Questionnaire (LSEQ),*[39,44] ten item questionnaire, self-rated, on 0-100 millimetre(mm) line. Validated tool for measuring changes in sleep on four domains; getting to sleep, quality of sleep, ease and awakening from sleep and alertness and behaviour following wakefulness.<br>The Dutch version of the LSEQ is forwards-and backwards translated according to the translation guidelines.[45]	x	x	
	Numeric rating scale for overall sleep, an overall numeric rating of quality of sleep. With '0' representing a very bad night's sleep and '10' representing a very good night's sleep.	x	x	
**Sleep pattern**	*The core consensus sleep diary,*[46] a ten item sleep log to gain insight in the sleep pattern of a participant.	x	x	
**Factor influencing sleep**	Based on literature,[15,47–50] an overview is made of ten influencing factors on sleep in hospitals. Participants can indicate whether these factors influenced their sleep and if so, to what extent.	x	x	
**Objective sleep parameters**	*Actigraphy, MotionWatch 8,* a wristwatch-like device, worn on the non-dominant hand. The actiwatch registers motion in epochs of 30 seconds. Based on motion sleep latency, sleep efficiency, number and duration of wake bouts, time awake after sleep onset in minutes can be calculated.	x	x	
**Cognition**	*Mini-Mental state Examination,*[51] A ten item screening tool to evaluate global cognitive function.	x		
	Stroop Colour-Word test,[52,53] consists of three subtasks. Used to measure attention, processing speed, cognitive flexibility and working memory.	x	x[1]	
	The Trail Making Test (TMT) A&B ,[54,55] consists of 2 subtasks, is used to measure visual search, attention, divided attention, processing speed and cognitive flexibility.	x	x[1]	
**Assessment of delirium**	*4 AT,*[56] A four item screening tool to detect attention deficits due to delirium.	x	x[2]	
	DSM-V,[57] the Diagnostic and statistical Manual of the American Psychiatric Association (DSM-5) the most widely used nomenclature by clinicians and researchers for the classification of delirium.	x[3]	x[3]	
**Severity of sleep problems**	*Insomnia severity index,*[58] a seven item questionnaire, self-rated, to quantify perceived insomnia severity.	x		
**Depression and anxiety**	*Hospital Anxiety Depression scale (HADS),*[59] a 14-item questionnaire, self-rated, consisting of two seven item subscales looking at depression and anxiety.	x		
**Level of independence**	*Katz-15 ADL index scale,*[60,61] the modified Katz ADL index 15 item-scale, answered with 'yes' or 'no' to evaluate the level of independence.	x		
**Health status**	*Rotterdam symptom check-list (RSCL),*[62] a 30 item questionnaire, self-rated, to assess physical and psychological symptom.	x	x[4]	
	Charlson comorbidity index (CCI),[63] a classification method for comorbid conditions. It is a valid method to estimate risk of death comorbid disease in one year.			x
**Vulnerability (e.g. fall risk)**	Safety management system patient screening *(VMS).*[64]	x		
**Adverse events**	Confusion, delirium, reduced consciousness, falls, aspiration, other additional adverse events (in complication register) and length of hospital stay in days.		x	

Table 1 Overview of instruments and timing of measurements

1. On day three Stroop Colour-Wordtest and TMT are repeated. 2. Only administered if a delirium is suspected. 3. When score on 4AT deviates, detailed assessment of mental status with DSM-V will be done. 4. On day four RSCL is repeated.

For peer review only - http://bmjopen.bmj.com/site/about/guidelines.xhtml

collected at baseline assessment. Demographic data consists of age, sex, nationality, highest educational status, living situation, marital status, height, weight, alcohol use and smoking habits. The recorded blood tests will include haemoglobin, leukocytes, C-reactive protein, electrolytes, kidney function, glucose and liver enzymes. To monitor adverse events there is a daily inquiry, for confusion, delirium, reduced consciousness, falls, aspiration and other additional adverse events. At discharge, medication use, diagnosis for hospitalisation and length of hospital stay in days will be recorded. To ensure consistency and optimisation of the data collection process, all members of the research team who will include patients in the study will follow a training how to conduct measurements according to Standard Operating Procedures (SOP) and how to fill out the Case Report Forms (CRF). Non-adherence to the study protocol will be obviated as much as possible. However, when it occurs this will be registered by the research team.

Sample size calculation

Sample size calculation is based on the primary outcome measure quality of sleep (QOS), assessed with the LSEQ. To establish a clinically relevant observed effect of melatonin, temazepam or placebo a difference of 10 mm on the QOS was chosen.[41] The standard deviation was calculated based on the standard error presented in this study. To detect a mean difference of 10 mm, two-sided, with a power of 80% and significance level of $\alpha < 0{,}05$, we need at least 210 patients per group (total of 630 patients).

The expected dropout rate during study period is 5%.[42] Therefore, we should recruit at least 663 patients, with 221 patients in each study arm.

Data analysis

Data will be analysed according to an intention-to-treat (ITT) principle. Researchers are not aware of the allocation of the intervention and therefore analyses will be conducted in a blinded way. A per-protocol analysis will be conducted excluding patients that did not receive the intervention according to protocol and were incorrectly included in the study. To determine the effects of temazepam, melatonin or placebo on sleep quality, linear mixed models will be used. Analysis will be performed on change of sleep quality over time taking baseline value into account (i.e. night before start of study medication). The following covariates will be added to the model: age, gender, different concurrent medication (e.g. morphine), severity of disease diagnosis (RSCL), pain severity (VAS-scale , RSCL), emergency or elective admission to the hospital, foregoing sleep problems (ISI), number of patients in the room, patients sleeping near a window, ADL independency (KATZ-ADL), level of anxiety (HADS) and days in the hospital before inclusion. Measurements used are presented in table

For peer review only - http://bmjopen.bmj.com/site/about/guidelines.xhtml

1. Sensitivity analysis will be conducted in order to determine the robustness of the results with regard to timing of measurement (3 versus 7 versus 10 days).

Linear mixed models will also be used to evaluate getting to sleep, awakening from sleep, behaviour following wakefulness and the objective sleep parameters measured with actigraphy. Analyses of the time needed to reach improvement of sleep quality will be done with an interval-censored survival analysis. Proportion of good nights of sleep measured with the numeric ratings scale will be analysed, when normally distributed, with multiple linear regression analysis. For the analysis of adverse events related to study medication, descriptive statistics will be used to show which adverse events occurred. Depending on the occurrence of these adverse events and distribution, log-linear analysis, Poisson or multiple regression analysis will be used. Time to occurrence of an adverse event will be analysed with Cox regression.

Data management

All participants are assigned a specific trial number, and all data will be entered in an electronic trial-specific database. The data entry process will be documented and produce an audit trail. The database will be stored and maintained by Castor EDC compliant with GCP guidelines[35] and the European Data Protection Directive. Confidentiality of participant information will be maintained throughout the trial. Results can only be traced to participants by the researchers involved. Data will be stored for a period of 15 years after the study has ended, according to ICH-GCP regulations.[35]

Participant safety and monitoring

The MATCH-trial is considered a low-risk study. Melatonin in a dosage of 1 mg is not known to show any adverse events and is considered safe for use by older patients. Temazepam has potential adverse-effects but this medication is part of usual treatment of hospitalised older patients with sleep problems, participation in the study will not expose patients to any additional risks. Close monitoring of all possible adverse events related to study medication will take place, and in case any adverse events occur, appropriate measures will be taken in consultation with the attending physician. The Clinical Research Unit (CRU) of the AMC will conduct the monitoring of this trial in compliance with the Medical Research Involving Human Subjects Act (WMO) and ICH-GCP regulations.[35] Annual reports of the study progress will be sent to the Medical Research Ethics Committee of the Academic Medical Centre Amsterdam.

For peer review only - http://bmjopen.bmj.com/site/about/guidelines.xhtml

ETHICS AND DISSEMINATION

This study was approved by the Medical Research Ethics Committee of the Academic Medical Centre Amsterdam in June 2017. Reference number: 2015_302.The executive boards of the other participating centres have provided local feasibility approval. The trial will be conducted in accordance with the Declaration of Helsinki 1996 and principles of good clinical practice. Any substantial protocol amendments will be submitted to the ethics committee. A register of protocol amendments will be made available in the study protocol. The grant was an unrestricted grant from the University of Amsterdam Foundation. Trial methods and results will be reported according to the CONSORT 2010 guidelines.[43]

Trial status

The trial has started on 29th of January 2018.

Information on trial and research group

Acknowledgements: The authors thank Carolien Jansen (data manager) and the senior citizens of Denktank 60+ Noord for their time, critical appraisal and advise.

Authors contributions: FS and SB contributed equally to this paper. SR is responsible for the original conception of the study and obtaining the grant. FS, SB, SJB, NS, BvM, NV, LH, SR attributed to the design of the study. FS and SB wrote the first draft of the manuscript, all authors contributed to further drafts. All authors read and approved the final draft.

Funding: The MATCH-trial is unrestrictedly financed by the Dalderup foundation.

Competing Interests: The authors declare no competing interests.

For peer review only

REFERENCES

1 Enomoto M, Tsutsui T, Higashino S, *et al.* Sleep-related problems and use of hypnotics in inpatients of acute hospital wards. *Gen Hosp Psychiatry* 2010;**32**:276–83. doi:10.1016/j.genhosppsych.2010.01.015

2 Isaia G, Corsinovi L, Bo M, *et al.* Insomnia among hospitalized elderly patients: prevalence, clinical characteristics and risk factors. *Arch Gerontol Geriatr* 2011;**52**:133–7. doi:10.1016/j.archger.2010.03.001

3 Meissner HH, Riemer A, Santiago SM, *et al.* Failure of physician documentation of sleep complaints in hospitalized patients. *West J Med* 1998;**169**:146–9.

4 Irwin MR, McClintick J, Costlow C, *et al.* Partial night sleep deprivation reduces natural killer and cellular immune responses in humans. *FASEB J* 1996;**10**:643–53.

5 Weinhouse GL. When Perception May Not Meet Reality : Sleep and. Crit. Care Med. 2014;**43**:248–9. doi:10.1097/CCM.0000000000000678

6 Inouye SK, Westendorp RGJ, Saczynski JS. Delirium in elderly people. *Lancet* 2014;**383**:911–22. doi:10.1016/S0140-6736(13)60688-1

7 Watson PL, Ceriana P, Fanfulla F. Delirium: is sleep important? *Best Pract Res Clin Anaesthesiol* 2012;**26**:355–66. doi:10.1016/j.bpa.2012.08.005

8 Chen S, Shi LG, Liang F, *et al.* Exogenous Melatonin for Delirium Prevention: a Meta-analysis of Randomized Controlled Trials. *Mol Neurobiol* 2016;**53**:4046–53. doi:10.1007/s12035-015-9350-8

9 Wu L, Sun D. Sleep duration and falls: a systemic review and meta-analysis of observational studies. *J Sleep Res* 2017;**26**:293–301. doi:10.1111/jsr.12505

10 Leproult R, Copinschi G, Buxton O, *et al.* Sleep loss results in an elevation of cortisol levels the next evening. *Sleep* 1997;**20**:865–70.

11 Kamdar BB, Needham DM, Collop NA. Sleep deprivation in critical illness: its role in physical and psychological recovery. *J Intensive Care Med* 2012;**27**:97–111. doi:10.1177/0885066610394322

12 Nederlande Internisten vereniging. Evidence-based richtlijn nieuwe of verergerende slaapproblemen bij acuut opgenomen ouderen in het ziekenhuis of verpleeghuis. Richtlijn. 2015;:1–89.https://internisten.nl/sites/internisten.nl/files/uploads/Me/i7/Mei7UYMgdwrpvUW8cZWRgQ/Richtlijn-NIV-Slaapproblemen---Geautoriseerde-versie.pdf (accessed 4 Nov 2017).

13 Young JS, Bourgeois JA, Hilty DM, *et al.* Sleep in hospitalized medical patients, Part 2: Behavioral and pharmacological management of sleep disturbances. *J Hosp Med* 2009;**4**:50–9.

For peer review only

doi:10.1002/jhm.397

14 Lenhart SE, Buysse DJ. Treatment of insomnia in hospitalized patients. *Ann Pharmacother* 2001;**35**:1449–57. doi:10.1345/aph.1A040

15 Tamrat R, Huynh-Le M-P, Goyal M. Non-Pharmacologic Interventions to Improve the Sleep of Hospitalized Patients: A Systematic Review. *J Gen Intern Med* 2014;**29**:788. doi:10.1007/S11606-013-2640-9

16 Kanji S, Mera A, Hutton B, *et al.* Pharmacological interventions to improve sleep in hospitalised adults: a systematic review. *BMJ Open* 2016;**6**:e012108. doi:10.1136/bmjopen-2016-012108

17 Frighetto L, Marra C, Bandali S, *et al.* An assessment of quality of sleep and the use of drugs with sedating properties in hospitalized adult patients. *Health Qual Life Outcomes* 2004;**2**:17. doi:10.1186/1477-7525-2-17

18 Gillis CM, Poyant JO, Degrado JR, *et al.* Inpatient pharmacological sleep aid utilization is common at a tertiary medical center. J. Hosp. Med. 2014;**9**:652–7. doi:10.1002/jhm.2246

19 Glass. Sedative hypnotics in older people with insomnia:meta-analysis of risk and benefits. *BMJ* 2005.

20 Cumming RG, Le Couteur DG. Benzodiazepines and risk of hip fractures in older people: a review of the evidence. *CNS Drugs* 2003;**17**:825–37.

21 Brandt J, Leong C. Benzodiazepines and Z-Drugs: An Updated Review of Major Adverse Outcomes Reported on in Epidemiologic Research. *Drugs R D* Published Online First: 1 September 2017. doi:10.1007/s40268-017-0207-7

22 Halfens R, Cox K, Kuppen???Van Merwijk A. Effect of the use of sleep medication in Dutch hospitals on the use of sleep medication at home. J. Adv. Nurs. 1994;**19**:66–70. doi:10.1111/j.1365-2648.1994.tb01052.x

23 Buscemi N, Vandermeer B, Hooton N, *et al.* The efficacy and safety of exogenous melatonin for primary sleep disorders. A meta-analysis. *J Gen Intern Med* 2005;**20**:1151–8. doi:10.1111/j.1525-1497.2005.0243.x

24 de Jonghe A, Korevaar JC, van Munster BC, *et al.* Effectiveness of melatonin treatment on circadian rhythm disturbances in dementia. Are there implications for delirium? A systematic review. *Int J Geriatr Psychiatry* 2010;**25**:1201–8. doi:10.1002/gps.2454

25 Chakraborti D, Tampi DJ, Tampi RR. Melatonin and Melatonin Agonist for Delirium in the Elderly Patients. *Am J Alzheimer's Dis Other Dementiasr* 2015;**30**:119–29. doi:10.1177/1533317514539379

26 Chen S, Shi L, Liang F, *et al.* Exogenous Melatonin for Delirium Prevention: a Meta-analysis of Randomized Controlled Trials. *Mol Neurobiol* 2016;**53**:4046–53. doi:10.1007/s12035-015-9350-8

For peer review only

27 Brzezinski A. Melatonin in humans.pdf. *N Engl J Med* 1997;**336**:186–95. doi:10.1056/NEJM199701163360306

28 Wade AG, Ford I, Crawford G, *et al.* Efficacy of prolonged release melatonin in insomnia patients aged 55-80 years: quality of sleep and next-day alertness outcomes. *Curr Med Res Opin* 2007;**23**:2597–605. doi:10.1185/030079907X233098

29 Lemoine P, Nir T, Laudon M, *et al.* Prolonged-release melatonin improves sleep quality and morning alertness in insomnia patients aged 55 years and older and has no withdrawal effects. *J Sleep Res* 2007;**16**:372–80. doi:10.1111/j.1365-2869.2007.00613.x

30 Bourne RS, Mills GH, Minelli C. Melatonin therapy to improve nocturnal sleep in critically ill patients: encouraging results from a small randomised controlled trial. *Crit Care* 2008;**12**:R52. doi:10.1186/cc6871

31 Shilo L, Dagan Y, Smorjik Y, *et al.* Effect of melatonin on sleep quality of COPD intensive care patients: a pilot study. *Chronobiol Int* 2000;**17**:71–6.

32 Ibrahim MG, Bellomo R, Hart GK, *et al.* A double-blind placebo-controlled randomised pilot study of nocturnal melatonin in tracheostomised patients. *Crit Care Resusc* 2006;**8**:187–91.

33 Andrade C, Srihari BS, Reddy KP, *et al.* Melatonin in medically ill patients with insomnia: a double-blind, placebo-controlled study. *J Clin Psychiatry* 2001;**62**:41–5.

34 Chan A-W, Tetzlaff JM, Altman DG, *et al*. SPIRIT 2013 Statement: Defining standard protocol items for clinical trials. Ann Intern Med. 2013;158(3):200-207

35 ICH. Integrated addendum to ICH e6(R1): Guideline for good clinical practice ICH. 2016.

36 Vural EMS, van Munster BC, de Rooij SE. Optimal dosages for melatonin supplementation therapy in older adults: a systematic review of current literature. *Drugs Aging* 2014;**31**:441–51. doi:10.1007/s40266-014-0178-0

37 Zorginstituut Nederland. Temazepam benzodiazepine agonisten | N05CD07. https://www.farmacotherapeutischkompas.nl/bladeren/preparaatteksten/t/temazepam (accessed 4 Nov 2017).

38 Parrott AC, Hindmarch I. The Leeds Sleep Evaluation Questionnaire in psychopharmacological investigations - a review. *Psychopharmacology (Berl)* 1980;**71**:173–9.

39 Tarrasch R, Laudon M, Zisapel N. Cross-cultural validation of the Leeds sleep evaluation questionnaire (LSEQ) in insomnia patients. *Hum Psychopharmacol* 2003;**18**:603–10. doi:10.1002/hup.534

40 Van Beuningen J, van der Houwen K, Moonen L. Measuring well-being An analysis of different response scales. The Hague/Heerlen: 2014.

41 Zisapel N, Nir T. Determination of the minimal clinically significant difference on a patient visual analog sleep quality scale. *J Sleep Res* 2003;**12**:291–8.

For peer review only

42 de Jonghe A, van Munster BC, Goslings JC, *et al.* Effect of melatonin on incidence of delirium among patients with hip fracture: a multicentre, double-blind randomized controlled trial. *CMAJ* 2014;**186**:E547-56. doi:10.1503/cmaj.140495

43 Moher D, Hopewell S, Schulz KF, *et al.* CONSORT 2010 explanation and elaboration: Updated guidelines for reporting parallel group randomised trials. *Int J Surg* 2012;**10**:28–55. doi:10.1016/j.ijsu.2011.10.001

44 Parrott AC, Hindmarch I. Factor analysis of a sleep evaluation questionnaire. *PsycholMed* 1978;**8**:325–9. doi:10.1017/S0033291700014379

45 Beaton DE, Bombardier C, Guillemin F, *et al.* Guidelines for the process of cross-cultural adaptation of self-report measures. *Spine (Phila Pa 1976)* 2000;**25**:3186–91.

46 Carney CE, Buysse DJ, Ancoli-Israel S, *et al.* The consensus sleep diary: standardizing prospective sleep self-monitoring. *Sleep* 2012;**35**:287–302. doi:10.5665/sleep.1642

47 Park MJ, Kim KH. What affects the subjective sleep quality of hospitalized elderly patients? *Geriatr Gerontol Int* 2016;:n/a-n/a. doi:10.1111/ggi.12743

48 Bano M, Chiaromanni F, Corrias M, *et al.* The Influence of Environmental Factors on Sleep Quality in Hospitalized Medical Patients. *Front Neurol* 2014;**5**:267. doi:10.3389/fneur.2014.00267

49 Ersser S, Wiles A, Taylor H, *et al.* The sleep of older people in hospital and nursing homes. *J Clin Nurs* 1999;**8**:360–8.

50 Dobing S, Frolova N, McAlister F, *et al.* Sleep quality and factors influencing self-reported sleep duration and quality in the general internal medicine inpatient population. *PLoS One* 2016;**11**:1–6. doi:10.1371/journal.pone.0156735

51 Folstein MF, Folstein SE, McHugh PR. 'Mini-mental state'. A practical method for grading the cognitive state of patients for the clinician. *J Psychiatr Res* 1975;**12**:189–98.

52 Stroop JR. Studies of interference in serial verbal reactions. *J Exp Psychol* 1935;**XVIII**:643–62.

53 Hammes J. De stroop Kleur-woord test: handleiding [The Stroop Color-word test: manual]. Lisse: 1997.

54 Army Individual Test Battery. Manual of Directions and Scoring. *Washington, DC War Dep Adjut Gen Off* 1944.

55 M.D. Lezak DBH. *Neuropsychological assessment*. 4th ed. New York: : Oxford University Press 2004.

56 Bellelli G, Morandi A, Davis DHJ, *et al.* Validation of the 4AT, a new instrument for rapid delirium screening: a study in 234 hospitalised older people. *Age Ageing* 2014;**43**:496–502. doi:10.1093/ageing/afu021

57 American Psychiatric Association. *Diagnostic and statistical manual of mental disorders*. 5th ed. Washington, DC: 2013.

For peer review only

58 Bastien CH, Vallières A, Morin CM. Validation of the Insomnia Severity Index as an outcome measure for insomnia research. *Sleep Med* 2001;**2**:297–307.

59 Cosco TD, Doyle F, Ward M, *et al.* Latent structure of the Hospital Anxiety And Depression Scale: a 10-year systematic review. *J Psychosom Res* 2012;**72**:180–4. doi:10.1016/j.jpsychores.2011.06.008

60 Laan W, Zuithoff NPA, Drubbel I, *et al.* Validity and reliability of the Katz-15 scale to measure unfavorable health outcomes in community-dwelling older people. *J Nutr Health Aging* 2014;**18**:848–54. doi:10.1007/s12603-014-0479-3

61 Weinberger M, Samsa GP, Schmader K, *et al.* Comparing proxy and patients' perceptions of patients' functional status: results from an outpatient geriatric clinic. *J Am Geriatr Soc* 1992;**40**:585–8.

62 de Haes JC, van Knippenberg FC, Neijt JP. Measuring psychological and physical distress in cancer patients: structure and application of the Rotterdam Symptom Checklist. *Br J Cancer* 1990;**62**:1034–8. doi:10.1038/bjc.1990.434

63 Charlson ME, Pompei P, Ales KL, *et al.* A new method of classifying prognostic comorbidity in longitudinal studies: development and validation. *J Chronic Dis* 1987;**40**:373–83.

64 Oud FMM, de Rooij SEJA, Schuurman T, *et al.* [Predictive value of the VMS theme 'Frail elderly': delirium, falling and mortality in elderly hospital patients]. *Ned Tijdschr Geneeskd* 2015;**159**:A8491.

For peer review only

Figure 1 Summary of MATCH trial

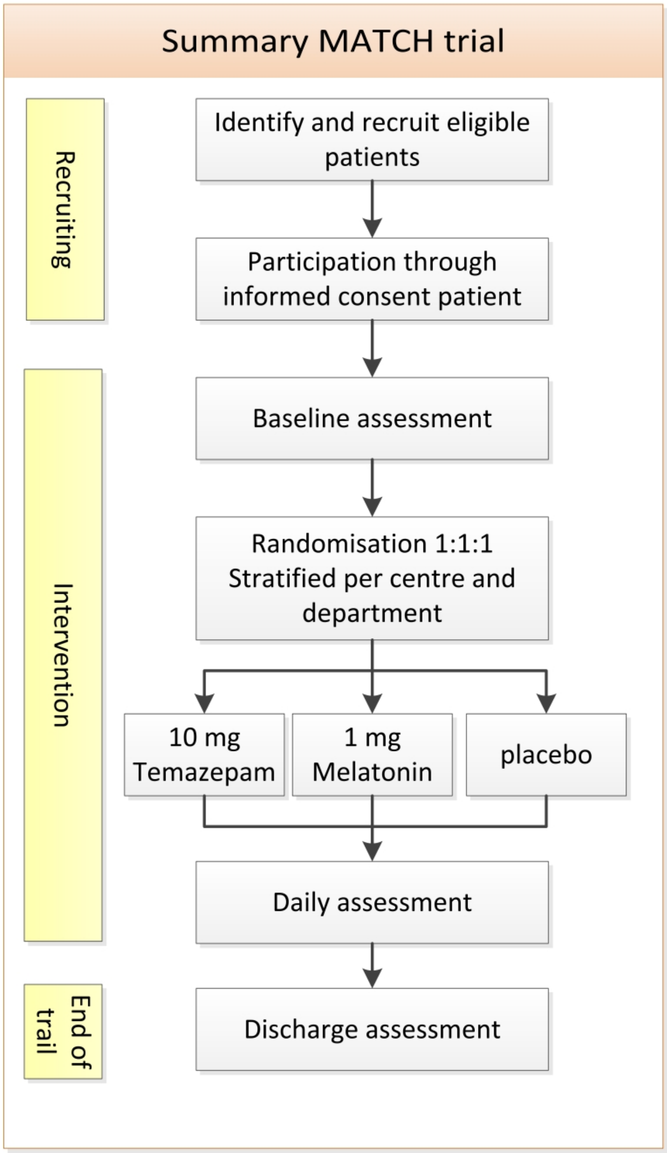

Figure 1. Flow chart of trial design summary

Reporting checklist for protocol of a clinical trial.

Based on the SPIRIT guidelines.

Instructions to authors

Complete this checklist by entering the page numbers from your manuscript where readers will find each of the items listed below.

Your article may not currently address all the items on the checklist. Please modify your text to include the missing information. If you are certain that an item does not apply, please write "n/a" and provide a short explanation.

Upload your completed checklist as an extra file when you submit to a journal.

In your methods section, say that you used the SPIRIT reporting guidelines, and cite them as:

Chan A-W, Tetzlaff JM, Altman DG, Laupacis A, Gøtzsche PC, Krleža-Jerić K, Hróbjartsson A, Mann H, Dickersin K, Berlin J, Doré C, Parulekar W, Summerskill W, Groves T, Schulz K, Sox H, Rockhold FW, Rennie D, Moher D. SPIRIT 2013 Statement: Defining standard protocol items for clinical trials. Ann Intern Med. 2013;158(3):200-207

		Reporting Item	Page Number
Title	#1	Descriptive title identifying the study design, population, interventions, and, if applicable, trial acronym	1
Trial registration	#2a	Trial identifier and registry name. If not yet registered, name of intended registry	2
Trial registration: data set	#2b	All items from the World Health Organization Trial Registration Data Set	See note 1
Protocol version	#3	Date and version identifier	1
Funding	#4	Sources and types of financial, material, and other support	10
Roles and responsibilities: contributorship	#5a	Names, affiliations, and roles of protocol contributors	1,10
Roles and	#5b	Name and contact information for the trial sponsor	See note 2

For peer review only

responsibilities: sponsor contact information			
Roles and responsibilities: sponsor and funder	#5c	Role of study sponsor and funders, if any, in study design; collection, management, analysis, and interpretation of data; writing of the report; and the decision to submit the report for publication, including whether they will have ultimate authority over any of these activities	See note 3
Roles and responsibilities: committees	#5d	Composition, roles, and responsibilities of the coordinating centre, steering committee, endpoint adjudication committee, data management team, and other individuals or groups overseeing the trial, if applicable (see Item 21a for data monitoring committee)	1,4
Background and rationale	#6a	Description of research question and justification for undertaking the trial, including summary of relevant studies (published and unpublished) examining benefits and harms for each intervention	2,3
Background and rationale: choice of comparators	#6b	Explanation for choice of comparators	3
Objectives	#7	Specific objectives or hypotheses	4
Trial design	#8	Description of trial design including type of trial (eg, parallel group, crossover, factorial, single group), allocation ratio, and framework (eg, superiority, equivalence, non-inferiority, exploratory)	4
Study setting	#9	Description of study settings (eg, community clinic, academic hospital) and list of countries where data will be collected. Reference to where list of study sites can be obtained	4
Eligibility criteria	#10	Inclusion and exclusion criteria for participants. If applicable, eligibility criteria for study centres and individuals who will perform the interventions (eg, surgeons, psychotherapists)	4,5
Interventions:	#11a	Interventions for each group with sufficient detail to allow	5,6

For peer review only

description		replication, including how and when they will be administered	
Interventions: modifications	#11b	Criteria for discontinuing or modifying allocated interventions for a given trial participant (eg, drug dose change in response to harms, participant request, or improving / worsening disease)	6
Interventions: adherance	#11c	Strategies to improve adherence to intervention protocols, and any procedures for monitoring adherence (eg, drug tablet return; laboratory tests)	See note 4
Interventions: concomitant care	#11d	Relevant concomitant care and interventions that are permitted or prohibited during the trial	5,6
Outcomes	#12	Primary, secondary, and other outcomes, including the specific measurement variable (eg, systolic blood pressure), analysis metric (eg, change from baseline, final value, time to event), method of aggregation (eg, median, proportion), and time point for each outcome. Explanation of the clinical relevance of chosen efficacy and harm outcomes is strongly recommended	6,7,8,9
Participant timeline	#13	Time schedule of enrolment, interventions (including any run-ins and washouts), assessments, and visits for participants. A schematic diagram is highly recommended (see Figure)	4 and figure 1
Sample size	#14	Estimated number of participants needed to achieve study objectives and how it was determined, including clinical and statistical assumptions supporting any sample size calculations	8
Recruitment	#15	Strategies for achieving adequate participant enrolment to reach target sample size	5
Allocation: sequence generation	#16a	Method of generating the allocation sequence (eg, computer-generated random numbers), and list of any factors for stratification. To reduce predictability of a random sequence, details of any planned restriction (eg, blocking) should be provided in a separate document that is unavailable to those who enrol participants or assign interventions	5

For peer review only

Allocation concealment mechanism	#16b	Mechanism of implementing the allocation sequence (eg, central telephone; sequentially numbered, opaque, sealed envelopes), describing any steps to conceal the sequence until interventions are assigned	5
Allocation: implementation	#16c	Who will generate the allocation sequence, who will enrol participants, and who will assign participants to interventions	5
Blinding (masking)	#17a	Who will be blinded after assignment to interventions (eg, trial participants, care providers, outcome assessors, data analysts), and how	5
Blinding (masking): emergency unblinding	#17b	If blinded, circumstances under which unblinding is permissible, and procedure for revealing a participant's allocated intervention during the trial	5
Data collection plan	#18a	Plans for assessment and collection of outcome, baseline, and other trial data, including any related processes to promote data quality (eg, duplicate measurements, training of assessors) and a description of study instruments (eg, questionnaires, laboratory tests) along with their reliability and validity, if known. Reference to where data collection forms can be found, if not in the protocol	6,7,8
Data collection plan: retention	#18b	Plans to promote participant retention and complete follow-up, including list of any outcome data to be collected for participants who discontinue or deviate from intervention protocols	See note 5
Data management	#19	Plans for data entry, coding, security, and storage, including any related processes to promote data quality (eg, double data entry; range checks for data values). Reference to where details of data management procedures can be found, if not in the protocol	9
Statistics: outcomes	#20a	Statistical methods for analysing primary and secondary outcomes. Reference to where other details of the statistical analysis plan can be found, if not in the protocol	8,9
Statistics: additional	#20b	Methods for any additional analyses (eg, subgroup and	8,9

For peer review only

analyses		adjusted analyses)	
Statistics: analysis population and missing data	#20c	Definition of analysis population relating to protocol non-adherence (eg, as randomised analysis), and any statistical methods to handle missing data (eg, multiple imputation)	See note 6
Data monitoring: formal committee	#21a	Composition of data monitoring committee (DMC); summary of its role and reporting structure; statement of whether it is independent from the sponsor and competing interests; and reference to where further details about its charter can be found, if not in the protocol. Alternatively, an explanation of why a DMC is not needed	9
Data monitoring: interim analysis	#21b	Description of any interim analyses and stopping guidelines, including who will have access to these interim results and make the final decision to terminate the trial	See note 7
Harms	#22	Plans for collecting, assessing, reporting, and managing solicited and spontaneously reported adverse events and other unintended effects of trial interventions or trial conduct	7, 8
Auditing	#23	Frequency and procedures for auditing trial conduct, if any, and whether the process will be independent from investigators and the sponsor	n/a
Research ethics approval	#24	Plans for seeking research ethics committee / institutional review board (REC / IRB) approval	10
Protocol amendments	#25	Plans for communicating important protocol modifications (eg, changes to eligibility criteria, outcomes, analyses) to relevant parties (eg, investigators, REC / IRBs, trial participants, trial registries, journals, regulators)	10
Consent or assent	#26a	Who will obtain informed consent or assent from potential trial participants or authorised surrogates, and how (see Item 32)	5
Consent or assent: ancillary studies	#26b	Additional consent provisions for collection and use of participant data and biological specimens in ancillary	n/a

For peer review only

		studies, if applicable	
Confidentiality	#27	How personal information about potential and enrolled participants will be collected, shared, and maintained in order to protect confidentiality before, during, and after the trial	9
Declaration of interests	#28	Financial and other competing interests for principal investigators for the overall trial and each study site	10
Data access	#29	Statement of who will have access to the final trial dataset, and disclosure of contractual agreements that limit such access for investigators	n/a, see note 3
Ancillary and post trial care	#30	Provisions, if any, for ancillary and post-trial care, and for compensation to those who suffer harm from trial participation	6
Dissemination policy: trial results	#31a	Plans for investigators and sponsor to communicate trial results to participants, healthcare professionals, the public, and other relevant groups (eg, via publication, reporting in results databases, or other data sharing arrangements), including any publication restrictions	2,10
Dissemination policy: authorship	#31b	Authorship eligibility guidelines and any intended use of professional writers	10
Dissemination policy: reproducible research	#31c	Plans, if any, for granting public access to the full protocol, participant-level dataset, and statistical code	n/a
Informed consent materials	#32	Model consent form and other related documentation given to participants and authorised surrogates	See note 8
Biological specimens	#33	Plans for collection, laboratory evaluation, and storage of biological specimens for genetic or molecular analysis in the current trial and for future use in ancillary studies, if applicable	n/a

Author notes

1. n/a see NTR registration. URL: http://www.trialregister.nl/trialreg/admin/rctview.asp?TC=6908
2. Universiteitsfonds UvA, contact information principal investigator (Professor Sophia E.de Rooij) page 1

For peer review only

3. n/a Investigator initiated, unrestricted (see page 10)

4. Patients are not admitted to the the hospital for sleeping problems, maximum duration of the study is 10 days. Although patients will often be discharged from the hospital before that.

5. n/a maximum of 10 study days (see also note 4)

6. 7 missing data n/a for this statistical method

7. n/a no interim analysis

8. n/a consent form approved by medical ethic commitee, the forms are in dutch and therefore not added

The SPIRIT checklist is distributed under the terms of the Creative Commons Attribution License CC-BY-ND 3.0. This checklist was completed on 29. June 2018 using https://www.goodreports.org/, a tool made by the EQUATOR Network in collaboration with Penelope.ai

For peer review only

BMJ Open

MelAtonin, Temazepam and plaCebo in Hospitalised older patients with sleeping problems (MATCH): a study protocol of randomised controlled trial

Journal:	*BMJ Open*
Manuscript ID	bmjopen-2018-025514.R1
Article Type:	Protocol
Date Submitted by the Author:	20-Dec-2018

Complete List of Authors:	Stenveld, Fiona; University of Groningen, University Medical Centre Groningen, Depatment of Geriatric Medicine<br>Bosman, Sjanne; University of Groningen, University Medical Centre Groningen, Department of Geriatric Medicine; Medical Centre Leeuwarden, Department of Geriatric Medicine<br>van Munster, Barbara; University of Groningen, University Medical Centre Groningen, Department of Geriatric Medicine; Gelre Hospitals, Department of Geriatrics<br>Beishuizen, Sara; Academic Medical Centre, Department of Internal Medicine, Geriatrics Section<br>Hempenius, Liesbeth; Medical Centre Leeuwarden, Department of Geriatric Medicine<br>van der Velde, Nathalie; Academic Medical Centre, Department of Internal Medicine, Geriatrics Section, Amsterdam Public Health<br>Smidt, Nynke; University of Groningen, University Medical Centre Groningen, Department of Geriatric Medicine; University of Groningen, University Medical Centre, Department of Epidemiology<br>De Rooij, Sophia; University of Groningen, University Medical Centre Groningen, Department of Geriatric Medicine; Academic Medical Centre, Department of Internal Medicine, Geriatrics Section, Amsterdam Public Health

**Primary Subject Heading**:	Geriatric medicine
Secondary Subject Heading:	Pharmacology and therapeutics, Evidence based practice
Keywords:	hospitalisation, aged, melatonin, benzodiazepines, sleep, randomised controlled trail

SCHOLARONE™
Manuscripts

Version 4.0, date 4-12-2018

MelAtonin, Temazepam and plaCebo in Hospitalised older patients with sleeping problems (MATCH): a study protocol of randomised controlled trial.

Fiona Stenveld(1)*, Sjanne Bosman*(1,2), Barbara C van Munster (1,3), Sara J.E. Beishuizen (4), Liesbeth Hempenius(2), Nathalie van der Velde (5), Nynke Smidt (1,6), Sophia E de Rooij (1,5)

Author affiliation:

1. University of Groningen, University Medical Centre Groningen, Department of Geriatric Medicine, Groningen, The Netherlands
2. Department of Geriatric Medicine, Medical Centre Leeuwarden, Leeuwarden, The Netherlands.
3. Department of Geriatrics, Gelre Hospitals, Apeldoorn, The Netherlands.
4. Department of Geriatrics, MC Slotervaart, Amsterdam
5. Department of Internal Medicine, Geriatrics Section, Academic Medical Centre, Amsterdam Public Health, Amsterdam, The Netherlands.
6. University of Groningen, University Medical Centre, Department of Epidemiology, Groningen, The Netherlands.

Correspondence public queries: s.bosman@umcg.nl, University Centre of Geriatric Medicine, University Medical Centre Groningen. Hanzeplein 1, PO box 30 001, 9700 RB Groningen

Correspondence scientific queries: Principal investigator: Professor Sophia E.de Rooij, s.e.j.a.de.rooij@umcg.nl telephone number 050-3613921, Hanzeplein 1, Postbus 30 001, 9700 RB Groningen. University of Groningen, University Medical Centre Groningen, University Centre for Geriatric Medicine, Groningen, The Netherlands.

Word count: 3828 (excluding title page, abstract, references, figures and tables)

For peer review only

ABSTRACT

Introduction: Hospitalised older patients frequently suffer from inadequate sleep, which can lead to patient distress and delayed recovery from acute illness or surgical procedure. Currently, no evidence-based treatments exist for sleeping problems in hospitalised older patients. Benzodiazepines, such as temazepam, are regularly prescribed by physicians, although they have serious side effects; for older patients in particular. Melatonin is proposed as a safe alternative for sleeping problems in hospitalised older patients, but the efficacy of melatonin is unclear in this population. Therefore, the aim of this study is to investigate the effects of melatonin and temazepam compared to placebo on sleep quality among hospitalised older patients with sleeping problems.

Methods and analysis: This study is a multicentre, randomised, placebo controlled trial. A total of 663 patients will be randomised in a 1:1:1 fashion to receive either melatonin (n=221), temazepam (n=221) or placebo (n=221). The study population consists of hospitalised patients aged 60 years and older, with new or aggravated sleeping problems for which an intervention is needed. The primary outcome is sleep quality measured with the Leeds Sleep Evaluation Questionnaire (LSEQ). Secondary outcomes include sleep parameters measured with actigraphy and medication-related adverse effects.

Ethics and dissemination: This study was approved by the Medical Ethics Committee of the Academic Medical Centre Amsterdam, (No 2015_302). Study findings will be disseminated through presentations at professional and scientific conferences and publications in peer-reviewed journals.

Trial registration number: NTR6908

Keywords: hospitalisation; aged; melatonin; benzodiazepines; temazepam; sleep; sleep disorders; insomnia; randomised controlled trial; actigraphy; adverse events.

Strengths and limitations of this study

- The results of this study will provide knowledge relevant for clinical practice and fill gaps in current guidelines concerning sleep problems in hospitalised older patients.
- To optimize the generalisability of the findings, this is a multicentre study with participants from different wards and minimal exclusions criteria.
- This study investigates, next to the effects, also the adverse events of melatonin and temazepam compared to placebo.
- Patients with cognitive impairment are excluded due to ethical considerations, even though these vulnerable patients may benefit the most from the study outcomes.

For peer review only

INTRODUCTION

Background

Hospitalised older patients frequently experience sleeping problems due to medical -, psychological- or environmental disturbances. The prevalence of sleep problems amongst older patients in medical and surgical wards varies between 30 and 65%[1–3]. Apart from the feeling of tiredness, insufficient sleep has substantial consequences for hospitalised patients. This includes delayed recovery from acute illness or surgical procedures because of its immunosuppressive effect[4,5], risk of delirium and falls[6–9] and disturbances in metabolic and endocrine functioning[10,11].

Thus, adequate treatment of sleeping problems is necessary. Today, it is still unclear how sleeping problems should be treated in the hospital. Non-pharmacological interventions are advised as first choice therapy in hospitalised patients[12–14], but there is limited evidence to support the effectiveness of non-pharmacological interventions[15]. With regard to pharmacological interventions, there is insufficient evidence to support its effectiveness for improving quality or quantity of sleep in hospitalised patients suffering from poor sleep[16].

Currently, up to 29% of older hospitalised patients with sleeping problems receive benzodiazepines[17,18], despite a recent meta-analysis of risks and benefits for short-term treatment of insomnia, which stated that benzodiazepines have little effect and may not outweigh the risks of adverse events, especially in a high risk older population[19]. Older patients have a higher risk of adverse drug events from benzodiazepines due to altered pharmacokinetics and pharmacodynamics. Common adverse drug events include in-hospital falls, delirium, aspiration, and respiratory depression [20,21]. Furthermore, use of benzodiazepine during hospitalisation can lead to prolonged use at home, which might promote chronic use and substance-dependency[22].

The neuroendocrine hormone melatonin has been proposed as a safer alternative to improve sleep quality because of its limited side effects[23–26]. Melatonin, produced by the pineal gland at night, is well known to influence circadian rhythm and sleep[27]. Two randomised control trials (RCT's) investigated the effectiveness of melatonin compared to placebo in older community dwelling persons with chronic complaints of insomnia and found that melatonin improved sleep quality significantly[28,29]. However, studies on the effectiveness of melatonin for sleeping problems in acute hospitalised older patients are scarce. Three RCT's have been performed in adult patients on the intensive care unit (ICU)[30–32], showing inconsistent results; one out of three reported a positive effect of melatonin[31]. All three studies were underpowered and none specifically assessed older patients with predefined sleep related problems.

For peer review only

Only one small RCT investigated the effectiveness of melatonin on a general medical ward in adults, which resulted in beneficial effects for melatonin compared to placebo on the sleep onset latency and patient satisfaction[33].

The overall aim of this study is to establish an evidence-based pharmacological treatment for hospitalised older patients with sleeping problems. In this study, we will firstly hypothesize that melatonin and temazepam are more effective than placebo on sleep quality among hospitalised older patients with sleeping problems and secondly melatonin is more effective than temazepam.

Objectives

The primary objective is to investigate firstly the effects of melatonin and temazepam compared to placebo among hospitalised older patients on self-reported sleep quality. Secondly, to investigate if melatonin is more effective than temazepam.

The secondary objectives are to investigate the effects of melatonin, temazepam and placebo on:

1. Other self-reported sleep parameters: getting to sleep, awakening from sleep, behaviour following wakefulness.
2. Objective sleep parameters: reduction in sleep onset latency, sleep efficiency, number and duration of wake bouts, time awake after sleep onset.
3. Adverse events related to study medication: Incidence of delirium, cognition, number of falls, complications.
4. Length of hospital stay in days.
5. Time needed to reach improvement in self-reported sleep quality
6. Proportion of good nights of sleep during study period.

METHODS

Trial design

This study is a multicentre, randomised, placebo-controlled trial. Figure 1 shows the study design of the MATCH-study. This study will be conducted in two university teaching hospitals and in two teaching hospitals in the Netherlands: the Academic Medical Centre (AMC) Amsterdam, the University Medical Centre Groningen (UMCG), Gelre Hospitals Apeldoorn, and the Medical Centre Leeuwarden (MCL). The design of this trial protocol has followed the recommendations of the SPIRIT 2013 statement[34].

For peer review only

Patient and public involvement

Senior citizens were involved in several stages of the is project, including the relevance of the study, developing the research question, the study design and conduct of the trial. The older representatives (Denktank 60+) partnered with us on the information material, the questionnaires and the burden of the questionnaires from a patient perspective. Once the trial has been published, participants will be informed of the results in a study newsletter suitable for the public.

Eligibility criteria

Eligible participants must have the following characteristics:

1. 60 years or older.
2. Admitted to the hospital for a medical or surgical reason.
3. Experiencing or complaining about new onset or aggravated sleep problems during hospital admission, for which a pharmacological intervention is needed based on the judgement of the attending physician.
4. Ability to fill out a sleep questionnaire.

People with the following characteristics are not eligible:

1. Inability to speak, understand or write Dutch.
2. Lack of decision making capacity.
3. Previously diagnosed dementia.
4. Transferred from another hospital to one of the study centres, with insufficient information on previous use of sleep medication.
5. Expected stay in hospital of <48 hours.
6. Concurrent use of sedative for a sleeping disorder (>2 dosages in the last week) , such as benzodiazepine, non-benzodiazepine (Z-drugs) or melatonin.
7. Alcohol consumption >13 units/week for women and >20 units/week for men.
8. Drug interactions with melatonin or contraindications for benzodiazepine use.

First selection of eligible patients will be done by the attending physician, if eligible the physician will inform the research team. Final selection will be done by a member of the research team, who also will recruit eligible participants and include them after written informed consent for participation has been obtained.

For peer review only

Randomisation and blinding

After patients have given informed consent, participants will be allocated randomly to either temazepam, melatonin or placebo capsules (ratio is 1:1:1). Randomisation is stratified per centre and department, the strata include the 4 centres (AMC, Gelre hospitals Apeldoorn, MCL and UMCG) and departments (surgical versus medical). A stratified block randomisation made by an independent statistician is used and generated by the program CASTOR Electronic Data Capture (Castor Electronic Data Capture, Ciwit BV, Amsterdam, the Netherlands, 2017). The researchers are not informed about the block sizes.

All medication used in the study will appear identical to ensure true blinding of the patient, attending physician, nurse and investigators. The study medication is manufactured by Tiofarma, Oud-Beijerland, the Netherlands and will be distributed to the study sites' pharmacies. Study medication must be kept intact at all times; the capsules cannot be broken. All study medication is packed in Single Unit Supply packages, blinded and dispensed by site trial pharmacists. The dispensing pharmacist will record the patient, patient ID and assigned medication by the randomisation process. Storage, delivery, dispensing and destruction of medication will adhere to ICH-GCP regulations[35].

At the end of the study, when all data is collected, the data manager will lock the database. The information of the study arm (melatonin, temazepam and placebo) will be masked by the data manager, after which analysis can be performed in a blinded way by the researchers. Randomisation method will be broken when the study team has declared the data analysis finished.

In case of emergencies or urgent situations, such as anaphylactic reactions or loss of consciousness, and knowledge of the actual treatment is essential for further patient management, the blinding of the treatment of an individual participant can be broken (unblinded). In this case, the attending physician will contact the trial pharmacist to disclose the blinding of the study treatment for this individual patient.

For peer review only

Intervention

After randomisation, all participants receive the allocated medication once daily which is in the evening before sleep (ante noctem). Study medication will be administered orally starting on the day of study enrolment.

Melatonin 1 mg and temazepam 10 mg will be used as study medication. The dosage of 1 mg melatonin is chosen based on a recent systematic review, to best mimic the normal physiological circadian rhythm of melatonin by avoiding prolonged supra-physiological blood levels in case of higher dosages[36]. The dosage of 10mg temazepam is the recommend dose for older patients[37].

For peer review only - http://bmjopen.bmj.com/site/about/guidelines.xhtml

Study medication will be continued to either a maximum of ten days, or 12 hours before hospital discharge, or after three consecutive nights of good sleep, or until participant decides to withdraw from the study. In case sleep problems do not resolve with study medication, the dosage can be doubled. This decision will be left to the patient's attending physician. Attending physicians are instructed only to consider a double dose when the patient has slept badly for two nights in a row, in case there are no environmental or treatable physical factors that could cause the poor quality of sleep and they should take possible adverse effects of study medication into account. Double doses of study medication will be registered in the case report form and in the medical record.

Study medication will be discontinued if a condition occurs that needs treatment with benzodiazepines (e.g. anxiety), if events occur that imply a higher risk of adverse events (e.g. respiratory insufficiency or starting chinolones) and in case of persisting sleeping problems. Persisting sleep problems is defined as self-reported poor quality of sleep for three consecutive nights after study medication has been doubled.

After study medication is discontinued, either due to a reason listed above or due to end of study period, treatment will be according to the hospital's protocol and under the responsibility of the attending physician. Patients remain in the study and follow the study protocol, except for using study medication.

Timing of measurement and outcome measures

Demographic data, such as age, sex, nationality, highest educational status and living situation will be collected at baseline. Medication use, blood tests 24 hours prior to study inclusion or 24 hours after study inclusion, severity of foregoing sleep problems, depression and anxiety, level of independence, health status and vulnerability (e.g. fall risk) are measured at baseline. Primary and secondary outcome measures will be recorded at baseline (T0) and every day until the 10th day (T1-T10) or until discharge. Table 1 provides an overview of the timing of measurements and the outcome measures.

Primary outcome:

The primary outcome of the study is change in self-reported sleep quality during the treatment period (T0-T10). Sleep quality will be assessed with the Leeds Sleep Evaluation Questionnaire (LSEQ)[38,39].

Secondary outcomes:

1. Change in self-reported sleep measures: getting to sleep, awakening from sleep and behaviour following wakefulness during treatment period will be assessed with the other subscales of the LSEQ. This measures will be assessed on T0 until T10.

For peer review only

2. Objective sleep parameters. To assess objective sleep parameters participants will wear a MotionWatch 8 during the study period (T0-T10). This wristwatch-like device will calculate sleep latency, sleep efficiency, number and duration of wake bouts, time awake after sleep onset in minutes based on motion of the patient.
3. Number of good night sleep. Good nights of sleep will be assessed with a numeric rating scale for quality of sleep. On the numeric rating scale ≤ 4 is considered a bad night's sleep, 5-6 is neither a good or bad night's sleep and ≥ 7 is a good night's sleep [40].
4. Time needed to reach improvement in self-reported sleep quality will be based on the LSEQ as well. Improvement of sleep quality is defined as the first day that an improvement of 10mm is reached compared to baseline.
5. Adverse events defined as confusion, delirium, reduced consciousness, falls, aspiration and other additional adverse events will be monitored daily on T0-T10.
6. Length of hospital stay in days will be recorded at discharge.

To ensure consistency and optimisation of the data collection process, all members of the research team who will include patients in the study will follow a training how to conduct measurements according to Standard Operating Procedures (SOP) and how to fill out the Case Report Forms (CRF). Non-adherence to the study protocol will be obviated as much as possible. However, when it occurs this will be registered by the research team.

Sample size calculation

Sample size calculation is based on the primary outcome measure quality of sleep (QOS), assessed with the LSEQ. To establish a clinically relevant observed effect of melatonin, temazepam or placebo a difference of 10 mm on the QOS was chosen. [41]. The standard deviation was calculated based on the standard error presented in this study. To detect a mean difference of 10 mm, two-sided, with a power of 80% and significance level of $\alpha < 0,05$, we need at least 210 patients per group (total of 630 patients).

The expected dropout rate during study period is 5% [42]. Therefore, we should recruit at least 663 patients, with 221 patients in each study arm.

Data analysis

Data will be analysed according to an intention-to-treat (ITT) principle. Researchers are not aware of the allocation of the intervention and therefore analyses will be conducted in a blinded way. A per-protocol analysis will be conducted excluding patients that did not receive the intervention according to protocol and were incorrectly included in the study. To determine the effects of temazepam, melatonin or placebo on sleep quality, linear mixed models will be used. Analysis will be performed

For peer review only

on change of sleep quality over time taking baseline value into account (i.e. night before start of study medication). The following covariates will be added to the model: age, gender, different concurrent medication (e.g. morphine), severity of disease diagnosis (RSCL), pain severity (VAS-scale , RSCL), acute or elective admission to the hospital, foregoing sleep problems (ISI), number of patients in the room, ADL independency (KATZ-ADL), level of anxiety (HADS) and days in the hospital before inclusion. Linear mixed models will also be used to evaluate getting to sleep, awakening from sleep, behaviour following wakefulness and the objective sleep parameters measured with actigraphy. Analyses of the time needed to reach improvement of sleep quality will be done with an interval-censored survival analysis. Proportion of good nights of sleep measured with the numeric ratings scale will be analysed, when normally distributed, with multiple linear regression analysis. For the analysis of adverse events related to study medication, descriptive statistics will be used to show which adverse events occurred. Depending on the occurrence of these adverse-events and distribution, log-linear analysis, Poisson or multiple regression analysis will be used. Time to occurrence of an adverse event will be analysed with Cox regression.

Data management

All participants are assigned a specific trial number, and all data will be entered in an electronic trial-specific database. The data entry process will be documented and produce an audit trail. The database will be stored and maintained by Castor EDC compliant with GCP guidelines [35] and the European Data Protection Directive. Confidentiality of participant information will be maintained throughout the trial. Results can only be traced to participants by the researchers involved. Data will be stored for a period of 15 years after the study has ended, according to ICH-GCP regulations[35].

Participant safety and monitoring

The MATCH-trial is considered a low-risk study. Melatonin in a dosage of 1 mg is not known to show any adverse events and is considered safe for use by older patients. Temazepam has potential adverse-effects but this medication is part of usual treatment of hospitalised older patients with sleep problems, participation in the study will not expose patients to any additional risks. Close monitoring of all possible adverse events related to study medication will take place, and in case any adverse events occur, appropriate measures will be taken in consultation with the attending physician. The Clinical Research Unit (CRU) of the AMC will conduct the monitoring of this trial in compliance with the Medical Research Involving Human Subjects Act (WMO) and ICH-GCP regulations[35]. Annual reports of the study progress will be sent to the Medical Research Ethics Committee of the Academic Medical Centre Amsterdam. As this trial is considered as a low risk study

For peer review only

with a low risk on serious adverse events, no Data and Safety Monitoring Board (DSMB) will be installed or interim analysis performed.

For peer review only

Measurement	Description and instrument	Timepoints		
		baseline	daily	discharge
**Sleep quality**	*Leeds Sleep Evaluation Questionnaire (LSEQ)*[39,43], ten item questionnaire, self-rated, on 0-100 millimetre(mm) line. Validated tool for measuring changes in sleep on four domains; getting to sleep, quality of sleep, ease and awakening from sleep and alertness and behaviour following wakefulness. The Dutch version of the LSEQ is forwards-and backwards translated according to the translation guidelines[44].	x	x	
	Numeric rating scale for overall sleep, an overall numeric rating of quality of sleep. With '0' representing a very bad night's sleep and '10' representing a very good night's sleep.	x	x	
**Sleep pattern**	*The core consensus sleep diary*[45]*,* a ten item sleep log to gain insight in the sleep pattern of a participant.	x	x	
**Factor influencing sleep**	Based on literature[15,46–49], an overview is made of ten influencing factors on sleep in hospitals. Participants can indicate whether these factors influenced their sleep and if so, to what extent.	x	x	
**Objective sleep parameters**	*Actigraphy, MotionWatch 8,* a wristwatch-like device, worn on the non-dominant hand. The actiwatch registers motion in epochs of 30 seconds. Based on motion sleep latency, sleep efficiency, number and duration of wake bouts, time awake after sleep onset in minutes can be calculated.	x	x	
**Cognition**	*Mini-Mental state Examination*[50]*,* A ten item screening tool to evaluate global cognitive function.	x		
	Stroop Colour-Word test[51,52]*,* consists of three subtasks. Used to measure attention, processing speed, cognitive flexibility and working memory.	x	x[1]	
	The Trail Making Test (TMT) A&B [53,54], consists of 2 subtasks, is used to measure visual search, attention, divided attention, processing speed and cognitive flexibility.	x	x[1]	
**Assessment of delirium**	*4 AT,* [55] A four item screening tool to detect attention deficits due to delirium.	x	x[2]	
	DSM-V,[56] the Diagnostic and statistical Manual of the American Psychiatric Association (DSM-5) the most widely used nomenclature by clinicians and researchers for the classification of delirium.	x[3]	x[3]	
**Severity of sleep problems**	*Insomnia severity index*[57], a seven item questionnaire, self-rated, to quantify perceived insomnia severity.	x		
**Depression and anxiety**	*Hospital Anxiety Depression scale (HADS)*[58], a 14-item questionnaire, self-rated, consisting of two seven item subscales looking at depression and anxiety.	x		
**Level of independence**	*Katz-15 ADL index scale,*[59,60] the modified Katz ADL index 15 item-scale, answered with 'yes' or 'no' to evaluate the level of independence.	x		
**Health status**	*Rotterdam symptom check-list (RSCL)*[61]*,* a 30 item questionnaire, self-rated, to assess physical and psychological symptom.	x	x[4]	
	Charlson comorbidity index (CCI),[62] a classification method for comorbid conditions. It is a valid method to estimate risk of death comorbid disease in one year.			x
**Vulnerability (e.g. fall risk)**	Safety management system patient screening *(VMS)*[63]	x		
**Adverse events**	Confusion, delirium, reduced consciousness, falls, aspiration, other additional adverse events (in complication register) and length of hospital stay in days.		x	

Table 1 Overview of the timing of measurements and outcome measures

1. On day three Stroop Colour-Wordtest and TMT are repeated. 2. Only administered if a delirium is suspected. 3. When score on 4AT deviates, detailed assessment of mental status with DSM-V will be done. 4. On day four RSCL is repeated.

For peer review only - http://bmjopen.bmj.com/site/about/guidelines.xhtml

DISCUSSION

Although sleeping problems are a common complaint in hospitalised older patients, there is still no evidence based pharmacological treatment available. Most studies on (non-)pharmacological treatment of sleeping problems in older patients concern studies in community dwelling older people. However, sleep quality in hospitalised elderly is influenced by other factors than community dwelling older people and might require other therapeutic regimes[64]. For example, light and noise in the room influences the quality of sleep in hospitalised older patients and it is difficult to measure, subsequently standardize for these confounders on the ward. In order to minimize the effect of these potential confounders, we stratified randomisation, in which patients are randomised by hospitals and departments. In this way, the known and unknown confounders are randomised and equally distributed among the groups.

In this study it was not possible to measure sleep quality and quantity using polysomnography, which is the gold standard to measure sleep quality and quantity. Instead of polysomnography, we measured objective sleep parameters indirectly by using an actigraphy, in addition to the self-reported sleep outcome measures.

The MATCH study is the first large randomised controlled trial on the effects of melatonin, temazepam or placebo in hospitalised older patients, which aimed to establish an evidence based pharmacological treatment for sleeping problems in older hospitalised patients. Irrespective of the results of this trial, the outcomes can be used for developing clinical guidelines for the treatment of sleep problems among hospitalized older patients.

ETHICS AND DISSEMINATION

This study was approved by the Medical Research Ethics Committee of the Academic Medical Centre Amsterdam in June 2017. Reference number: 2015_302.The executive boards of the other participating centres have provided local feasibility approval. The trial will be conducted in accordance with the Declaration of Helsinki 1996 and principles of good clinical practice. Any substantial protocol amendments will be submitted to the ethics committee. A register of protocol amendments will be made available in the study protocol.

The grant was an unrestricted grant from the University of Amsterdam Foundation.

Trial methods and results will be reported according to the CONSORT 2010 guidelines[65]. The study findings will be published in relevant peer-reviewed journals.

Trial status

For peer review only

The trial has started on 29th of January 2018.

Information on trial and research group

Acknowledgements: The authors thank Carolien Jansen (data manager) and the senior citizens of Denktank 60+ Noord for their time, critical appraisal and advise.

Authors contributions: FS and SB contributed equally to this paper. SR is responsible for the original conception of the study and obtaining the grant. FS, SB, SJB, NS, BvM, NV, LH, SR attributed to the design of the study. FS and SB wrote the first draft of the manuscript, all authors contributed to further drafts. All authors read and approved the final draft.

Funding: The MATCH-trial is unrestrictedly financed by the Dalderup foundation. Competing Interests: The authors declare no competing interests.

For peer review only

REFERENCES

1 Enomoto M, Tsutsui T, Higashino S, *et al.* Sleep-related problems and use of hypnotics in inpatients of acute hospital wards. *Gen Hosp Psychiatry* 2010;**32**:276–83. doi:10.1016/j.genhosppsych.2010.01.015

2 Isaia G, Corsinovi L, Bo M, *et al.* Insomnia among hospitalized elderly patients: prevalence, clinical characteristics and risk factors. *Arch Gerontol Geriatr* 2011;**52**:133–7. doi:10.1016/j.archger.2010.03.001

3 Meissner HH, Riemer A, Santiago SM, *et al.* Failure of physician documentation of sleep complaints in hospitalized patients. *West J Med* 1998;**169**:146–9.http://www.pubmedcentral.nih.gov/articlerender.fcgi?artid=1305196&tool=pmcentrez&rendertype=abstract (accessed 17 Jan 2017).

4 Irwin MR, McClintick J, Costlow C, *et al.* Partial night sleep deprivation reduces natural killer and cellular immune responses in humans. *FASEB J* 1996;**10**:643–53.

5 Weinhouse GL. When Perception May Not Meet Reality : Sleep and. Crit. Care Med. 2014;**43**:248–9. doi:10.1097/CCM.0000000000000678

6 Inouye SK, Westendorp RGJ, Saczynski JS. Delirium in elderly people. *Lancet* 2014;**383**:911–22. doi:10.1016/S0140-6736(13)60688-1

7 Watson PL, Ceriana P, Fanfulla F. Delirium: is sleep important? *Best Pract Res Clin Anaesthesiol* 2012;**26**:355–66. doi:10.1016/j.bpa.2012.08.005

8 Chen S, Shi LG, Liang F, *et al.* Exogenous Melatonin for Delirium Prevention: a Meta-analysis of Randomized Controlled Trials. *Mol Neurobiol* 2016;**53**:4046–53. doi:10.1007/s12035-015-9350-8

9 Wu L, Sun D. Sleep duration and falls: a systemic review and meta-analysis of observational studies. *J Sleep Res* 2017;**26**:293–301. doi:10.1111/jsr.12505

10 Leproult R, Copinschi G, Buxton O, *et al.* Sleep loss results in an elevation of cortisol levels the next evening. *Sleep* 1997;**20**:865–70.http://www.ncbi.nlm.nih.gov/pubmed/9415946 (accessed 11 Aug 2017).

11 Kamdar BB, Needham DM, Collop NA. Sleep deprivation in critical illness: its role in physical and psychological recovery. *J Intensive Care Med* 2012;**27**:97–111. doi:10.1177/0885066610394322

12 Nederlande Internisten vereniging. Evidence-based richtlijn nieuwe of verergerende slaapproblemen bij acuut opgenomen ouderen in het ziekenhuis of verpleeghuis. Richtlijn. 2015;:1-89. https://internisten.nl/sites/internisten.nl/files/uploads/Me/i7/Mei7UYMgdwrpvUW8cZWRgQ

For peer review only

/Richtlijn-NIV-Slaapproblemen---Geautoriseerde-versie.pdf (accessed 4 Nov 2017).

13 Young JS, Bourgeois JA, Hilty DM, *et al.* Sleep in hospitalized medical patients, Part 2: Behavioral and pharmacological management of sleep disturbances. *J Hosp Med* 2009;**4**:50–9. doi:10.1002/jhm.397

14 Lenhart SE, Buysse DJ. Treatment of insomnia in hospitalized patients. *Ann Pharmacother* 2001;**35**:1449–57. doi:10.1345/aph.1A040

15 Tamrat R, Huynh-Le M-P, Goyal M. Non-Pharmacologic Interventions to Improve the Sleep of Hospitalized Patients: A Systematic Review. *J Gen Intern Med* 2014;**29**:788. doi:10.1007/S11606-013-2640-9

16 Kanji S, Mera A, Hutton B, *et al.* Pharmacological interventions to improve sleep in hospitalised adults: a systematic review. *BMJ Open* 2016;**6**:e012108. doi:10.1136/bmjopen-2016-012108

17 Frighetto L, Marra C, Bandali S, *et al.* An assessment of quality of sleep and the use of drugs with sedating properties in hospitalized adult patients. *Health Qual Life Outcomes* 2004;**2**:17. doi:10.1186/1477-7525-2-17

18 Gillis CM, Poyant JO, Degrado JR, *et al.* Inpatient pharmacological sleep aid utilization is common at a tertiary medical center. J. Hosp. Med. 2014;**9**:652–7. doi:10.1002/jhm.2246

19 Glass. Sedative hypnotics in older people with insomnia:meta-analysis of risk and benefits. *BMJ* 2005.

20 Cumming RG, Le Couteur DG. Benzodiazepines and risk of hip fractures in older people: a review of the evidence. *CNS Drugs* 2003;**17**:825–37.http://www.ncbi.nlm.nih.gov/pubmed/12921493 (accessed 18 Jan 2017).

21 Brandt J, Leong C. Benzodiazepines and Z-Drugs: An Updated Review of Major Adverse Outcomes Reported on in Epidemiologic Research. *Drugs R D* Published Online First: 1 September 2017. doi:10.1007/s40268-017-0207-7

22 Halfens R, Cox K, Kuppen???Van Merwijk A. Effect of the use of sleep medication in Dutch hospitals on the use of sleep medication at home. J. Adv. Nurs. 1994;**19**:66–70. doi:10.1111/j.1365-2648.1994.tb01052.x

23 Buscemi N, Vandermeer B, Hooton N, *et al.* The efficacy and safety of exogenous melatonin for primary sleep disorders. A meta-analysis. *J Gen Intern Med* 2005;**20**:1151–8. doi:10.1111/j.1525-1497.2005.0243.x

24 de Jonghe A, Korevaar JC, van Munster BC, *et al.* Effectiveness of melatonin treatment on circadian rhythm disturbances in dementia. Are there implications for delirium? A systematic review. *Int J Geriatr Psychiatry* 2010;**25**:1201–8. doi:10.1002/gps.2454

25 Chakraborti D, Tampi DJ, Tampi RR. Melatonin and Melatonin Agonist for Delirium in the Elderly Patients. *Am J Alzheimer's Dis Other Dementiasr* 2015;**30**:119–29.

For peer review only

doi:10.1177/1533317514539379

26 Chen S, Shi L, Liang F, *et al.* Exogenous Melatonin for Delirium Prevention: a Meta-analysis of Randomized Controlled Trials. *Mol Neurobiol* 2016;**53**:4046–53. doi:10.1007/s12035-015-9350-8

27 Brzezinski A. Melatonin in humans.pdf. *N Engl J Med* 1997;**336**:186–95. doi:10.1056/NEJM199701163360306

28 Wade AG, Ford I, Crawford G, *et al.* Efficacy of prolonged release melatonin in insomnia patients aged 55-80 years: quality of sleep and next-day alertness outcomes. *Curr Med Res Opin* 2007;**23**:2597–605. doi:10.1185/030079907X233098

29 Lemoine P, Nir T, Laudon M, *et al.* Prolonged-release melatonin improves sleep quality and morning alertness in insomnia patients aged 55 years and older and has no withdrawal effects. *J Sleep Res* 2007;**16**:372–80. doi:10.1111/j.1365-2869.2007.00613.x

30 Bourne RS, Mills GH, Minelli C. Melatonin therapy to improve nocturnal sleep in critically ill patients: encouraging results from a small randomised controlled trial. *Crit Care* 2008;**12**:R52. doi:10.1186/cc6871

31 Shilo L, Dagan Y, Smorjik Y, *et al.* Effect of melatonin on sleep quality of COPD intensive care patients: a pilot study. *Chronobiol Int* 2000;**17**:71–6.http://www.ncbi.nlm.nih.gov/pubmed/10672435 (accessed 18 Jan 2017).

32 Ibrahim MG, Bellomo R, Hart GK, *et al.* A double-blind placebo-controlled randomised pilot study of nocturnal melatonin in tracheostomised patients. *Crit Care Resusc* 2006;**8**:187–91.http://www.ncbi.nlm.nih.gov/pubmed/16930101 (accessed 18 Jan 2017).

33 Andrade C, Srihari BS, Reddy KP, *et al.* Melatonin in medically ill patients with insomnia: a double-blind, placebo-controlled study. *J Clin Psychiatry* 2001;**62**:41–5.http://www.ncbi.nlm.nih.gov/pubmed/11235927 (accessed 18 Jan 2017).

34 Agha RA, Altman DG, Rosin D. The SPIRIT 2013 statement – Defining standard protocol items for trials. *Int J Surg* 2015;**13**:288–91. doi:10.1016/j.ijsu.2014.12.007

35 ICH. Integrated addendum to ICH e6(R1): Guideline for good clinical practice ICH. 2016.

36 Vural EMS, van Munster BC, de Rooij SE. Optimal dosages for melatonin supplementation therapy in older adults: a systematic review of current literature. *Drugs Aging* 2014;**31**:441–51. doi:10.1007/s40266-014-0178-0

37 Zorginstituut Nederland. Temazepam benzodiazepine agonisten | N05CD07. https://www.farmacotherapeutischkompas.nl/bladeren/preparaatteksten/t/temazepam (accessed 4 Nov 2017).

38 Parrott AC, Hindmarch I. The Leeds Sleep Evaluation Questionnaire in psychopharmacological investigations - a review. *Psychopharmacology (Berl)* 1980;**71**:173–

For peer review only

9.http://www.ncbi.nlm.nih.gov/pubmed/6777817 (accessed 19 Jan 2017).

39 Tarrasch R, Laudon M, Zisapel N. Cross-cultural validation of the Leeds sleep evaluation questionnaire (LSEQ) in insomnia patients. *Hum Psychopharmacol* 2003;**18**:603–10. doi:10.1002/hup.534

40 Van Beuningen J, van der Houwen K, Moonen L. Measuring well-being An analysis of different response scales. The Hague/Heerlen: 2014. www.cbs.nl

41 Zisapel N, Nir T. Determination of the minimal clinically significant difference on a patient visual analog sleep quality scale. *J Sleep Res* 2003;**12**:291–8.http://www.ncbi.nlm.nih.gov/pubmed/14633240 (accessed 18 Jan 2017).

42 de Jonghe A, van Munster BC, Goslings JC, *et al.* Effect of melatonin on incidence of delirium among patients with hip fracture: a multicentre, double-blind randomized controlled trial. *CMAJ* 2014;**186**:E547-56. doi:10.1503/cmaj.140495

43 Parrott AC, Hindmarch I. Factor analysis of a sleep evaluation questionnaire. *PsycholMed* 1978;**8**:325–9. doi:10.1017/S0033291700014379

44 Beaton DE, Bombardier C, Guillemin F, *et al.* Guidelines for the process of cross-cultural adaptation of self-report measures. *Spine (Phila Pa 1976)* 2000;**25**:3186–91.http://www.ncbi.nlm.nih.gov/pubmed/11124735 (accessed 20 Aug 2017).

45 Carney CE, Buysse DJ, Ancoli-Israel S, *et al.* The consensus sleep diary: standardizing prospective sleep self-monitoring. *Sleep* 2012;**35**:287–302. doi:10.5665/sleep.1642

46 Park MJ, Kim KH. What affects the subjective sleep quality of hospitalized elderly patients? *Geriatr Gerontol Int* 2016;:n/a-n/a. doi:10.1111/ggi.12743

47 Bano M, Chiaromanni F, Corrias M, *et al.* The Influence of Environmental Factors on Sleep Quality in Hospitalized Medical Patients. *Front Neurol* 2014;**5**:267. doi:10.3389/fneur.2014.00267

48 Ersser S, Wiles A, Taylor H, *et al.* The sleep of older people in hospital and nursing homes. *J Clin Nurs* 1999;**8**:360–8.http://www.ncbi.nlm.nih.gov/pubmed/10624252 (accessed 13 Mar 2017).

49 Dobing S, Frolova N, McAlister F, *et al.* Sleep quality and factors influencing self-reported sleep duration and quality in the general internal medicine inpatient population. *PLoS One* 2016;**11**:1–6. doi:10.1371/journal.pone.0156735

50 Folstein MF, Folstein SE, McHugh PR. 'Mini-mental state'. A practical method for grading the cognitive state of patients for the clinician. *J Psychiatr Res* 1975;**12**:189–98.http://www.ncbi.nlm.nih.gov/pubmed/1202204 (accessed 21 Sep 2016).

51 Stroop JR. Studies of interference in serial verbal reactions. *J Exp Psychol* 1935;**XVIII**:643–62.

52 Hammes J. De stroop Kleur-woord test: handleiding [The Stroop Color-word test: manual]. Lisse: 1997.

53 Army Individual Test Battery. Manual of Directions and Scoring. *Washington, DC War Dep Adjut*

For peer review only

Gen Off 1944.

54 M.D. Lezak DBH. *Neuropsychological assessment*. 4th ed. New York: : Oxford University Press 2004.

55 Bellelli G, Morandi A, Davis DHJ, *et al.* Validation of the 4AT, a new instrument for rapid delirium screening: a study in 234 hospitalised older people. *Age Ageing* 2014;**43**:496–502. doi:10.1093/ageing/afu021

56 American Psychiatric Association. *Diagnostic and statistical manual of mental disorders*. 5th ed. Washington, DC: 2013.

57 Bastien CH, Vallières A, Morin CM. Validation of the Insomnia Severity Index as an outcome measure for insomnia research. *Sleep Med* 2001;**2**:297–307.http://www.ncbi.nlm.nih.gov/pubmed/11438246 (accessed 11 Aug 2017).

58 Cosco TD, Doyle F, Ward M, *et al.* Latent structure of the Hospital Anxiety And Depression Scale: a 10-year systematic review. *J Psychosom Res* 2012;**72**:180–4. doi:10.1016/j.jpsychores.2011.06.008

59 Laan W, Zuithoff NPA, Drubbel I, *et al.* Validity and reliability of the Katz-15 scale to measure unfavorable health outcomes in community-dwelling older people. *J Nutr Health Aging* 2014;**18**:848–54. doi:10.1007/s12603-014-0479-3

60 Weinberger M, Samsa GP, Schmader K, *et al.* Comparing proxy and patients' perceptions of patients' functional status: results from an outpatient geriatric clinic. *J Am Geriatr Soc* 1992;**40**:585–8.http://www.ncbi.nlm.nih.gov/pubmed/1587975 (accessed 19 Jan 2017).

61 de Haes JC, van Knippenberg FC, Neijt JP. Measuring psychological and physical distress in cancer patients: structure and application of the Rotterdam Symptom Checklist. *Br J Cancer* 1990;**62**:1034–8. doi:10.1038/bjc.1990.434

62 Charlson ME, Pompei P, Ales KL, *et al.* A new method of classifying prognostic comorbidity in longitudinal studies: development and validation. *J Chronic Dis* 1987;**40**:373–83.http://www.ncbi.nlm.nih.gov/pubmed/3558716 (accessed 25 Oct 2016).

63 Oud FMM, de Rooij SEJA, Schuurman T, *et al.* [Predictive value of the VMS theme 'Frail elderly': delirium, falling and mortality in elderly hospital patients]. *Ned Tijdschr Geneeskd* 2015;**159**:A8491.http://www.ncbi.nlm.nih.gov/pubmed/25761294 (accessed 4 Nov 2017).

64 Wesselius HM, Van Den Ende ES, Alsma J, *et al.* Quality and quantity of sleep and factors associated with sleep disturbance in hospitalized patients. *JAMA Intern Med* 2018;**178**:1165–71. doi:10.1001/jamainternmed.2018.2669

65 Moher D, Hopewell S, Schulz KF, *et al.* CONSORT 2010 explanation and elaboration: Updated guidelines for reporting parallel group randomised trials. *Int J Surg* 2012;**10**:28–55. doi:10.1016/j.ijsu.2011.10.001

For peer review only

Figure 1. Flow chart of trial design summary

For peer review only

Figure 1 Summary of MATCH trial

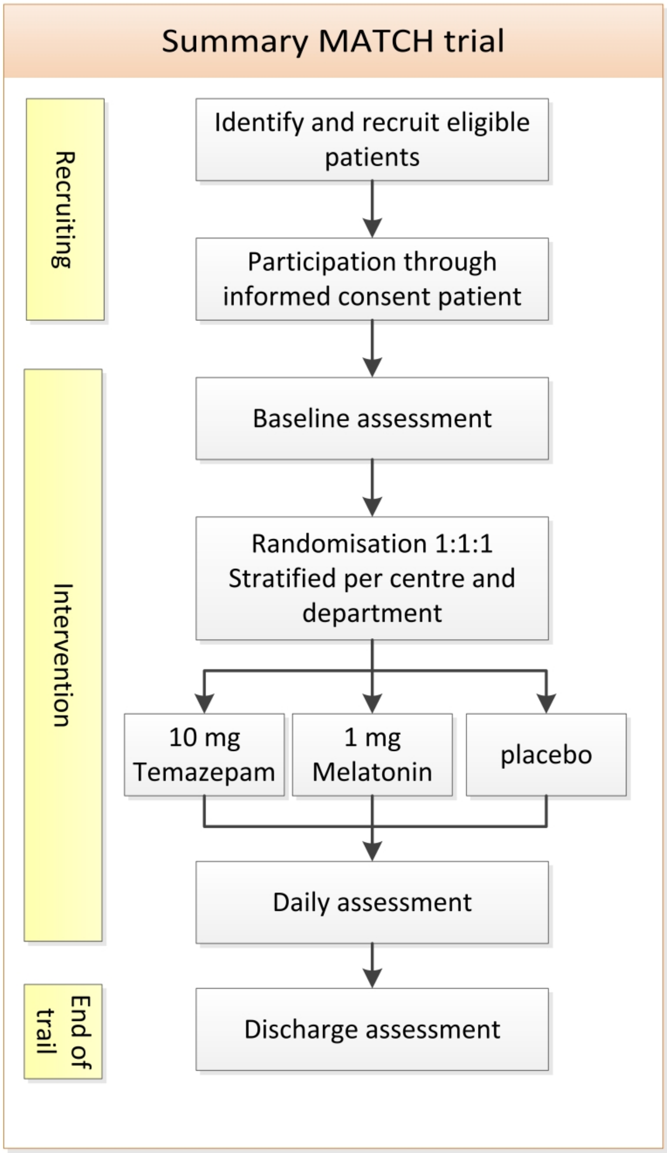

Figure 1. Flow chart of trial design summary

Reporting checklist for protocol of a clinical trial.

Based on the SPIRIT guidelines.

Instructions to authors

Complete this checklist by entering the page numbers from your manuscript where readers will find each of the items listed below.

Your article may not currently address all the items on the checklist. Please modify your text to include the missing information. If you are certain that an item does not apply, please write "n/a" and provide a short explanation.

Upload your completed checklist as an extra file when you submit to a journal.

In your methods section, say that you used the SPIRIT reporting guidelines, and cite them as:

Chan A-W, Tetzlaff JM, Altman DG, Laupacis A, Gøtzsche PC, Krleža-JeriĆ K, Hróbjartsson A, Mann H, Dickersin K, Berlin J, Doré C, Parulekar W, Summerskill W, Groves T, Schulz K, Sox H, Rockhold FW, Rennie D, Moher D. SPIRIT 2013 Statement: Defining standard protocol items for clinical trials. Ann Intern Med. 2013;158(3):200-207

		Reporting Item	Page Number
Title	#1	Descriptive title identifying the study design, population, interventions, and, if applicable, trial acronym	1
Trial registration	#2a	Trial identifier and registry name. If not yet registered, name of intended registry	2
Trial registration: data set	#2b	All items from the World Health Organization Trial Registration Data Set	See note 1
Protocol version	#3	Date and version identifier	1
Funding	#4	Sources and types of financial, material, and other support	12
Roles and responsibilities: contributorship	#5a	Names, affiliations, and roles of protocol contributors	1,12
Roles and responsibilities: sponsor contact information	#5b	Name and contact information for the trial sponsor	See note 2
Roles and	#5c	Role of study sponsor and funders, if any, in study design; collection, management,	See note 3

For peer review only

responsibilities: sponsor and funder		analysis, and interpretation of data; writing of the report; and the decision to submit the report for publication, including whether they will have ultimate authority over any of these activities	
Roles and responsibilities: committees	#5d	Composition, roles, and responsibilities of the coordinating centre, steering committee, endpoint adjudication committee, data management team, and other individuals or groups overseeing the trial, if applicable (see Item 21a for data monitoring committee)	1,4
Background and rationale	#6a	Description of research question and justification for undertaking the trial, including summary of relevant studies (published and unpublished) examining benefits and harms for each intervention	3
Background and rationale: choice of comparators	#6b	Explanation for choice of comparators	3
Objectives	#7	Specific objectives or hypotheses	4
Trial design	#8	Description of trial design including type of trial (eg, parallel group, crossover, factorial, single group), allocation ratio, and framework (eg, superiority, equivalence, non-inferiority, exploratory)	4
Study setting	#9	Description of study settings (eg, community clinic, academic hospital) and list of countries where data will be collected. Reference to where list of study sites can be obtained	4
Eligibility criteria	#10	Inclusion and exclusion criteria for participants. If applicable, eligibility criteria for study centres and individuals who will perform the interventions (eg, surgeons, psychotherapists)	5
Interventions: description	#11a	Interventions for each group with sufficient detail to allow replication, including how and when they will be administered	6,7
Interventions: modifications	#11b	Criteria for discontinuing or modifying allocated interventions for a given trial participant (eg, drug dose change in response to harms, participant request, or improving / worsening disease)	7
Interventions: adherance	#11c	Strategies to improve adherence to intervention protocols, and any procedures for monitoring adherence (eg, drug tablet return; laboratory tests)	See note 4
Interventions: concomitant care	#11d	Relevant concomitant care and interventions that are permitted or prohibited during the trial	6,7
Outcomes	#12	Primary, secondary, and other outcomes, including the specific measurement variable	7,8,9

For peer review only

		(eg, systolic blood pressure), analysis metric (eg, change from baseline, final value, time to event), method of aggregation (eg, median, proportion), and time point for each outcome. Explanation of the clinical relevance of chosen efficacy and harm outcomes is strongly recommended	
Participant timeline	#13	Time schedule of enrolment, interventions (including any run-ins and washouts), assessments, and visits for participants. A schematic diagram is highly recommended (see Figure)	7 and figure 1
Sample size	#14	Estimated number of participants needed to achieve study objectives and how it was determined, including clinical and statistical assumptions supporting any sample size calculations	8
Recruitment	#15	Strategies for achieving adequate participant enrolment to reach target sample size	5
Allocation: sequence generation	#16a	Method of generating the allocation sequence (eg, computer-generated random numbers), and list of any factors for stratification. To reduce predictability of a random sequence, details of any planned restriction (eg, blocking) should be provided in a separate document that is unavailable to those who enrol participants or assign interventions	6
Allocation concealment mechanism	#16b	Mechanism of implementing the allocation sequence (eg, central telephone; sequentially numbered, opaque, sealed envelopes), describing any steps to conceal the sequence until interventions are assigned	6
Allocation: implementation	#16c	Who will generate the allocation sequence, who will enrol participants, and who will assign participants to interventions	6
Blinding (masking)	#17a	Who will be blinded after assignment to interventions (eg, trial participants, care providers, outcome assessors, data analysts), and how	6
Blinding (masking): emergency unblinding	#17b	If blinded, circumstances under which unblinding is permissible, and procedure for revealing a participant's allocated intervention during the trial	6
Data collection plan	#18a	Plans for assessment and collection of outcome, baseline, and other trial data, including any related processes to promote data quality (eg, duplicate measurements, training of assessors) and a description of study instruments (eg, questionnaires, laboratory tests) along with their reliability and validity, if known. Reference to where data collection forms can be found, if not in the protocol	7,8,9,10
Data collection plan: retention	#18b	Plans to promote participant retention and complete follow-up, including list of any outcome data to be collected for participants who discontinue or deviate from intervention protocols	See note 5

For peer review only

Data management	#19	Plans for data entry, coding, security, and storage, including any related processes to promote data quality (eg, double data entry; range checks for data values). Reference to where details of data management procedures can be found, if not in the protocol	9
Statistics: outcomes	#20a	Statistical methods for analysing primary and secondary outcomes. Reference to where other details of the statistical analysis plan can be found, if not in the protocol	8,9
Statistics: additional analyses	#20b	Methods for any additional analyses (eg, subgroup and adjusted analyses)	8,9
Statistics: analysis population and missing data	#20c	Definition of analysis population relating to protocol non-adherence (eg, as randomised analysis), and any statistical methods to handle missing data (eg, multiple imputation)	See note 6
Data monitoring: formal committee	#21a	Composition of data monitoring committee (DMC); summary of its role and reporting structure; statement of whether it is independent from the sponsor and competing interests; and reference to where further details about its charter can be found, if not in the protocol. Alternatively, an explanation of why a DMC is not needed	9
Data monitoring: interim analysis	#21b	Description of any interim analyses and stopping guidelines, including who will have access to these interim results and make the final decision to terminate the trial	9
Harms	#22	Plans for collecting, assessing, reporting, and managing solicited and spontaneously reported adverse events and other unintended effects of trial interventions or trial conduct	8,9
Auditing	#23	Frequency and procedures for auditing trial conduct, if any, and whether the process will be independent from investigators and the sponsor	n/a
Research ethics approval	#24	Plans for seeking research ethics committee / institutional review board (REC / IRB) approval	11
Protocol amendments	#25	Plans for communicating important protocol modifications (eg, changes to eligibility criteria, outcomes, analyses) to relevant parties (eg, investigators, REC / IRBs, trial participants, trial registries, journals, regulators)	11
Consent or assent	#26a	Who will obtain informed consent or assent from potential trial participants or authorised surrogates, and how (see Item 32)	5
Consent or assent: ancillary studies	#26b	Additional consent provisions for collection and use of participant data and biological specimens in ancillary studies, if applicable	n/a
Confidentiality	#27	How personal information about potential and enrolled participants will be collected, shared, and maintained in order to protect confidentiality before, during, and after the	9

For peer review only

		trial	
Declaration of interests	#28	Financial and other competing interests for principal investigators for the overall trial and each study site	11
Data access	#29	Statement of who will have access to the final trial dataset, and disclosure of contractual agreements that limit such access for investigators	n/a, see note 3
Ancillary and post trial care	#30	Provisions, if any, for ancillary and post-trial care, and for compensation to those who suffer harm from trial participation	7
Dissemination policy: trial results	#31a	Plans for investigators and sponsor to communicate trial results to participants, healthcare professionals, the public, and other relevant groups (eg, via publication, reporting in results databases, or other data sharing arrangements), including any publication restrictions	2,11
Dissemination policy: authorship	#31b	Authorship eligibility guidelines and any intended use of professional writers	11
Dissemination policy: reproducible research	#31c	Plans, if any, for granting public access to the full protocol, participant-level dataset, and statistical code	n/a
Informed consent materials	#32	Model consent form and other related documentation given to participants and authorised surrogates	See note 8
Biological specimens	#33	Plans for collection, laboratory evaluation, and storage of biological specimens for genetic or molecular analysis in the current trial and for future use in ancillary studies, if applicable	n/a

Author notes

1. n/a see NTR registration. URL: http://www.trialregister.nl/trialreg/admin/rctview.asp?TC=6908
2. Universiteitsfonds UvA, contact information principal investigator (Professor Sophia E.de Rooij) page 1
3. n/a Investigator initiated, unrestricted (see page 10)
4. Patients are not admitted to the hospital for sleeping problems, maximum duration of the study is 10 days. Although patients will often be discharged from the hospital before that.
5. n/a maximum of 10 study days (see also note 4)
6. 7 missing data n/a for this statistical method
7. n/a no interim analysis

For peer review only

8. n/a consent form approved by medical ethic commitee, the forms are in dutch and therefore not added

The SPIRIT checklist is distributed under the terms of the Creative Commons Attribution License CC-BY-ND 3.0. This checklist was completed on 29. June 2018 using https://www.goodreports.org/, a tool made by the EQUATOR Network in collaboration with Penelope.ai

For peer review only

BMJ Open

MelAtonin, Temazepam and plaCebo in Hospitalised older patients with sleeping problems (MATCH): a study protocol of randomised controlled trial

Journal:	*BMJ Open*
Manuscript ID	bmjopen-2018-025514.R2
Article Type:	Protocol
Date Submitted by the Author:	14-Mar-2019
Complete List of Authors:	Stenveld, Fiona; University of Groningen, University Medical Centre Groningen, Depatment of Geriatric Medicine<br>Bosman, Sjanne; University of Groningen, University Medical Centre Groningen, Department of Geriatric Medicine; Medical Centre Leeuwarden, Department of Geriatric Medicine<br>van Munster, Barbara; University of Groningen, University Medical Centre Groningen, Department of Geriatric Medicine; Gelre Hospitals, Department of Geriatrics<br>Beishuizen, Sara; Academic Medical Centre, Department of Internal Medicine, Geriatrics Section<br>Hempenius, Liesbeth; Medical Centre Leeuwarden, Department of Geriatric Medicine<br>van der Velde, Nathalie; Academic Medical Centre, Department of Internal Medicine, Geriatrics Section, Amsterdam Public Health<br>Smidt, Nynke; University of Groningen, University Medical Centre Groningen, Department of Geriatric Medicine; University of Groningen, University Medical Centre, Department of Epidemiology<br>De Rooij, Sophia; University of Groningen, University Medical Centre Groningen, Department of Geriatric Medicine; Academic Medical Centre, Department of Internal Medicine, Geriatrics Section, Amsterdam Public Health
**Primary Subject Heading**:	Geriatric medicine
Secondary Subject Heading:	Pharmacology and therapeutics, Evidence based practice
Keywords:	hospitalisation, aged, melatonin, benzodiazepines, sleep, randomised controlled trail

SCHOLARONE™
Manuscripts

Version 5.0, date 07-03-2019

MelAtonin, Temazepam and plaCebo in Hospitalised older patients with sleeping problems (MATCH): a study protocol of randomised controlled trial.

Fiona Stenveld(1)*, Sjanne Bosman*(1,2), Barbara C van Munster (1,3), Sara J.E. Beishuizen (4), Liesbeth Hempenius(2), Nathalie van der Velde (5), Nynke Smidt (1,6), Sophia E de Rooij (1,5)

Author affiliation:

1. University of Groningen, University Medical Centre Groningen, Department of Geriatric Medicine, Groningen, The Netherlands
2. Department of Geriatric Medicine, Medical Centre Leeuwarden, Leeuwarden, The Netherlands.
3. Department of Geriatrics, Gelre Hospitals, Apeldoorn, The Netherlands.
4. Department of Geriatrics, MC Slotervaart, Amsterdam
5. Department of Internal Medicine, Geriatrics Section, Academic Medical Centre, Amsterdam Public Health, Amsterdam, The Netherlands.
6. University of Groningen, University Medical Centre, Department of Epidemiology, Groningen, The Netherlands.

Correspondence public queries: s.bosman@umcg.nl, University Centre of Geriatric Medicine, University Medical Centre Groningen. Hanzeplein 1, PO box 30 001, 9700 RB Groningen

Correspondence scientific queries: Principal investigator: Professor Sophia E.de Rooij, s.e.j.a.de.rooij@umcg.nl telephone number 050-3613921, Hanzeplein 1, Postbus 30 001, 9700 RB Groningen. University of Groningen, University Medical Centre Groningen, University Centre for Geriatric Medicine, Groningen, The Netherlands.

Word count: 3947 (excluding title page, abstract, references, figures and tables)

For peer review only

ABSTRACT

Introduction: Hospitalised older patients frequently suffer from inadequate sleep, which can lead to patient distress and delayed recovery from acute illness or surgical procedure. Currently, no evidence-based treatments exist for sleeping problems in hospitalised older patients. Benzodiazepines, such as temazepam, are regularly prescribed by physicians, although they have serious side effects; for older patients in particular. Melatonin is proposed as a safe alternative for sleeping problems in hospitalised older patients, but the efficacy of melatonin is unclear in this population. Therefore, the aim of this study is to investigate the effects of melatonin and temazepam compared to placebo on sleep quality among hospitalised older patients with sleeping problems.

Methods and analysis: This study is a multicentre, randomised, placebo controlled trial. A total of 663 patients will be randomised in a 1:1:1 fashion to receive either melatonin (n=221), temazepam (n=221) or placebo (n=221). The study population consists of hospitalised patients aged 60 years and older, with new or aggravated sleeping problems for which an intervention is needed. The primary outcome is sleep quality measured with the Leeds Sleep Evaluation Questionnaire (LSEQ). Secondary outcomes include sleep parameters measured with actigraphy and medication-related adverse effects.

Ethics and dissemination: This study was approved by the Medical Ethics Committee of the Academic Medical Centre Amsterdam, (No 2015_302). Study findings will be disseminated through presentations at professional and scientific conferences and publications in peer-reviewed journals.

Trial registration number: NTR6908

Keywords: hospitalisation; aged; melatonin; benzodiazepines; temazepam; sleep; sleep disorders; insomnia; randomised controlled trial; actigraphy; adverse events.

Strengths and limitations of this study

- The results of this study will provide knowledge relevant for clinical practice and fill gaps in current guidelines concerning sleep problems in hospitalised older patients.
- To optimize the generalisability of the findings, this is a multicentre study with participants from different wards and minimal exclusions criteria.
- This study investigates, next to the effects, also the adverse events of melatonin and temazepam compared to placebo.
- Patients with cognitive impairment are excluded due to ethical considerations, even though these vulnerable patients may benefit the most from the study outcomes.

For peer review only

INTRODUCTION

Background

Hospitalised older patients frequently experience sleeping problems due to medical -, psychological- or environmental disturbances. The prevalence of sleep problems amongst older patients in medical and surgical wards varies between 30 and 65%[1–3]. Apart from the feeling of tiredness, insufficient sleep has substantial consequences for hospitalised patients. This includes delayed recovery from acute illness or surgical procedures because of its immunosuppressive effect[4,5], risk of delirium and falls[6–9] and disturbances in metabolic and endocrine functioning[10,11].

Thus, adequate treatment of sleeping problems is necessary. Today, it is still unclear how sleeping problems should be treated in the hospital. Non-pharmacological interventions are advised as first choice therapy in hospitalised patients[12–14], but there is limited evidence to support the effectiveness of non-pharmacological interventions[15]. With regard to pharmacological interventions, there is insufficient evidence to support its effectiveness for improving quality or quantity of sleep in hospitalised patients suffering from poor sleep[16].

Currently, up to 29% of older hospitalised patients with sleeping problems receive benzodiazepines[17,18], despite a recent meta-analysis of risks and benefits for short-term treatment of insomnia, which stated that benzodiazepines have little effect and may not outweigh the risks of adverse events, especially in a high risk older population[19]. Older patients have a higher risk of adverse drug events from benzodiazepines due to altered pharmacokinetics and pharmacodynamics. Common adverse drug events include in-hospital falls, delirium, aspiration, and respiratory depression [20,21]. Furthermore, use of benzodiazepine during hospitalisation can lead to prolonged use at home, which might promote chronic use and substance-dependency[22].

The neuroendocrine hormone melatonin has been proposed as a safer alternative to improve sleep quality because of its limited side effects[23–26]. Melatonin, produced by the pineal gland at night, is well known to influence circadian rhythm and sleep[27]. Two randomised control trials (RCT's) investigated the effectiveness of melatonin compared to placebo in older community dwelling persons with chronic complaints of insomnia and found that melatonin improved sleep quality significantly[28,29]. However, studies on the effectiveness of melatonin for sleeping problems in acute hospitalised older patients are scarce. Three RCT's have been performed in adult patients on the intensive care unit (ICU)[30–32], showing inconsistent results; one out of three reported a positive effect of melatonin[31]. All three studies were underpowered and none specifically assessed older patients with predefined sleep related problems.

For peer review only

Only one small RCT investigated the effectiveness of melatonin on a general medical ward in adults, which resulted in beneficial effects for melatonin compared to placebo on the sleep onset latency and patient satisfaction[33].

The overall aim of this study is to establish an evidence-based pharmacological treatment for hospitalised older patients with sleeping problems. In this study, we will firstly hypothesize that melatonin and temazepam are more effective than placebo on sleep quality among hospitalised older patients with sleeping problems and secondly melatonin is more effective than temazepam.

Objectives

The primary objective is to investigate firstly the effects of melatonin and temazepam compared to placebo among hospitalised older patients on self-reported sleep quality. Secondly, to investigate if melatonin is more effective than temazepam.

The secondary objectives are to investigate the effects of melatonin, temazepam and placebo on:

1. Other self-reported sleep parameters: getting to sleep, awakening from sleep, behaviour following wakefulness.
2. Objective sleep parameters: reduction in sleep onset latency, sleep efficiency, number and duration of wake bouts, time awake after sleep onset.
3. Adverse events related to study medication: Incidence of delirium, cognition, number of falls, complications.
4. Length of hospital stay in days.
5. Time needed to reach improvement in self-reported sleep quality
6. Proportion of good nights of sleep during study period.

METHODS

Trial design

This study is a multicentre, randomised, placebo-controlled trial. Figure 1 shows the study design of the MATCH-study. This study will be conducted in two university teaching hospitals and in two teaching hospitals in the Netherlands: the Academic Medical Centre (AMC) Amsterdam, the University Medical Centre Groningen (UMCG), Gelre Hospitals Apeldoorn, and the Medical Centre Leeuwarden (MCL). The design of this trial protocol has followed the recommendations of the SPIRIT 2013 statement[34].

For peer review only

Patient and public involvement

Senior citizens were involved in several stages of this project, including the relevance of the study, developing the research question, the study design and conduct of the trial. The older representatives (Denktank 60+) partnered with us on the information material, the questionnaires and the burden of the questionnaires from a patient perspective. Once the trial has been published, participants will be informed of the results in a study newsletter suitable for the public.

Eligibility criteria

Eligible participants must have the following characteristics:

1. 60 years or older.
2. Admitted to the hospital for a medical or surgical reason.
3. Experiencing or complaining about new onset or aggravated sleep problems during hospital admission, for which a pharmacological intervention is needed based on the judgement of the attending physician.
4. Ability to fill out a sleep questionnaire.

People with the following characteristics are not eligible:

1. Inability to speak, understand or write Dutch.
2. Lack of decision making capacity.
3. Previously diagnosed dementia.
4. Transferred from another hospital to one of the study centres, with insufficient information on previous use of sleep medication.
5. Expected stay in hospital of <48 hours.
6. Concurrent use of sedative for a sleeping disorder (>2 dosages in the last week) , such as benzodiazepine, non-benzodiazepine (Z-drugs) or melatonin.
7. Alcohol consumption >13 units/week for women and >20 units/week for men.
8. Drug interactions with melatonin or contraindications for benzodiazepine use.

First selection of eligible patients will be done by the attending physician, if eligible the physician will inform the research team. Final selection will be done by a member of the research team, who also will recruit eligible participants and include them after written informed consent for participation has been obtained.

For peer review only

Randomisation and blinding

After patients have given informed consent, participants will be allocated randomly to either temazepam, melatonin or placebo capsules (ratio is 1:1:1). Randomisation is stratified per centre and department, the strata include the 4 centres (AMC, Gelre hospitals Apeldoorn, MCL and UMCG) and departments (surgical versus medical). A stratified block randomisation made by an independent statistician is used and generated by the program CASTOR Electronic Data Capture (Castor EDC) (Castor Electronic Data Capture, Ciwit BV, Amsterdam, the Netherlands, 2017). The researchers are not informed about the block sizes.

All medication used in the study will appear identical to ensure true blinding of the patient, attending physician, nurse and investigators. The study medication is manufactured by Tiofarma, Oud-Beijerland, the Netherlands and will be distributed to the study sites' pharmacies. Study medication must be kept intact at all times; the capsules cannot be broken. All study medication is packed in Single Unit Supply packages, blinded and dispensed by site trial pharmacists. The dispensing pharmacist will record the patient, patient ID and assigned medication by the randomisation process. Storage, delivery, dispensing and destruction of medication will adhere to Good Clinical Practice regulations (ICH-GCP)[35].

At the end of the study, when all data is collected, the data manager will lock the database. The information of the study arm (melatonin, temazepam and placebo) will be masked by the data manager, after which analysis can be performed in a blinded way by the researchers. Randomisation method will be broken when the study team has declared the data analysis finished.

In case of emergencies or urgent situations, such as anaphylactic reactions or loss of consciousness, and knowledge of the actual treatment is essential for further patient management, the blinding of the treatment of an individual participant can be broken (unblinded). In this case, the attending physician will contact the trial pharmacist to disclose the blinding of the study treatment for this individual patient.

For peer review only

Intervention

After randomisation, all participants receive the allocated medication once daily which is in the evening before sleep (ante noctem). Study medication will be administered orally starting on the day of study enrolment.

Melatonin 1 mg and temazepam 10 mg will be used as study medication. The dosage of 1 mg melatonin is chosen based on a recent systematic review, to best mimic the normal physiological circadian rhythm of melatonin by avoiding prolonged supra-physiological blood levels in case of higher dosages[36]. The dosage of 10mg temazepam is the recommend dose for older patients[37].

For peer review only - http://bmjopen.bmj.com/site/about/guidelines.xhtml

Study medication will be continued to either a maximum of ten days, or 12 hours before hospital discharge, or after three consecutive nights of good sleep, or until participant decides to withdraw from the study. In case sleep problems do not resolve with study medication, the dosage can be doubled. This decision will be left to the patient's attending physician. Attending physicians are instructed only to consider a double dose when the patient has slept badly for two nights in a row, in case there are no environmental or treatable physical factors that could cause the poor quality of sleep and they should take possible adverse effects of study medication into account. Double doses of study medication will be registered in the case report form and in the medical record.

Study medication will be discontinued if a condition occurs that needs treatment with benzodiazepines (e.g. anxiety), if events occur that imply a higher risk of adverse events (e.g. respiratory insufficiency or starting chinolones) and in case of persisting sleeping problems. Persisting sleep problems is defined as self-reported poor quality of sleep for three consecutive nights after study medication has been doubled.

After study medication is discontinued, either due to a reason listed above or due to end of study period, treatment will be provided according to the hospital's protocol and under the responsibility of the attending physician. Patients remain in the study and follow the study protocol, except for using study medication.

Timing of measurement and outcome measures

Demographic data, such as age, sex, nationality, highest educational status and living situation will be collected at baseline. Medication use, blood tests 24 hours prior to study inclusion or 24 hours after study inclusion, severity of foregoing sleep problems, depression and anxiety, level of independence, health status and vulnerability (e.g. fall risk) are measured at baseline. Primary and secondary outcome measures will be recorded at baseline (T0) and every day until the 10th day (T1-T10) or until discharge. Table 1 provides an overview of the timing of measurements and the outcome measures.

For peer review only

Primary outcome:

The primary outcome of the study is change in self-reported sleep quality during the treatment period (T0-T10). Sleep quality will be assessed with the Leeds Sleep Evaluation Questionnaire (LSEQ)[38,39].

Secondary outcomes:

1. Change in self-reported sleep measures: getting to sleep, awakening from sleep and behaviour following wakefulness during treatment period will be assessed with the other subscales of the LSEQ. This measures will be assessed on T0 until T10.

2. Objective sleep parameters. To assess objective sleep parameters participants will wear a MotionWatch 8 during the study period (T0-T10). This wristwatch-like device will calculate sleep latency, sleep efficiency, number and duration of wake bouts, time awake after sleep onset in minutes based on motion of the patient.
3. Number of good night sleep. Good nights of sleep will be assessed with a numeric rating scale for quality of sleep. On the numeric rating scale ≤ 4 is considered a bad night's sleep, 5-6 is neither a good or bad night's sleep and ≥ 7 is a good night's sleep [40].
4. Time needed to reach improvement in self-reported sleep quality will be based on the LSEQ as well. Improvement of sleep quality is defined as the first day that an improvement of 10mm is reached compared to baseline.
5. Adverse events defined as confusion, delirium, reduced consciousness, falls, aspiration and other additional adverse events will be monitored daily on T0-T10.
6. Length of hospital stay in days will be recorded at discharge.

To ensure consistency and optimisation of the data collection process, all members of the research team who will include patients in the study will follow a training how to conduct measurements according to Standard Operating Procedures (SOP) and how to fill out the Case Report Forms (CRF). Non-adherence to the study protocol will be obviated as much as possible. However, when it occurs this will be registered by the research team.

Sample size calculation

Sample size calculation is based on the primary outcome measure quality of sleep (QOS), assessed with the LSEQ. To establish a clinically relevant observed effect of melatonin, temazepam or placebo a difference of 10 mm on the QOS was chosen. [41]. The standard deviation was calculated based on the standard error presented in this study. To detect a mean difference of 10 mm, two-sided, with a power of 80% and significance level of α < 0,05, we need at least 210 patients per group (total of 630 patients).

The expected dropout rate during study period is 5% [42]. Therefore, we should recruit at least 663 patients, with 221 patients in each study arm.

Data analysis

Data will be analysed according to an intention-to-treat (ITT) principle. Researchers are not aware of the allocation of the intervention and therefore analyses will be conducted in a blinded way. A per-protocol analysis will be conducted excluding patients that did not receive the intervention according to protocol and were incorrectly included in the study. To determine the effects of temazepam, melatonin or placebo on sleep quality, linear mixed models will be used. Analysis will be performed

For peer review only

on change of sleep quality over time taking baseline value into account (i.e. night before start of study medication). The following covariates will be added to the model: age, gender, different concurrent medication (e.g. morphine), severity of disease diagnosis (Rotterdam Symptom Checklist (RSCL), pain severity (visual analogue scale(VAS) , RSCL), acute or elective admission to the hospital, foregoing sleep problems (Insomnia severity index (ISI), number of patients in the room, level of independency (Katz Index of Independence in Activities of Daily Living (KATZ-ADL), level of anxiety (Hospital Anxiety Depression scale (HADS) and days in the hospital before inclusion. Linear mixed models will also be used to evaluate getting to sleep, awakening from sleep, behaviour following wakefulness and the objective sleep parameters measured with actigraphy. Analyses of the time needed to reach improvement of sleep quality will be done with an interval-censored survival analysis. Proportion of good nights of sleep measured with the numeric ratings scale will be analysed, when normally distributed, with multiple linear regression analysis. For the analysis of adverse events related to study medication, descriptive statistics will be used to show which adverse events occurred. Depending on the occurrence of these adverse-events and distribution, log-linear analysis, Poisson or multiple regression analysis will be used. Time to occurrence of an adverse event will be analysed with Cox regression.

Data management

All participants are assigned a specific trial number, and all data will be entered in an electronic trial-specific database. The data entry process will be documented and produce an audit trail. The database will be stored and maintained by Castor EDC compliant with ICH-GCP regulations[35] and the European Data Protection Directive. Confidentiality of participant information will be maintained throughout the trial. Results can only be traced to participants by the researchers involved. Data will be stored for a period of 15 years after the study has ended, according to ICH-GCP regulations[35].

Participant safety and monitoring

The MATCH-trial is considered a low-risk study. Melatonin in a dosage of 1 mg is not known to show any adverse events and is considered safe for use by older patients. Temazepam has potential adverse-effects but this medication is part of usual treatment of hospitalised older patients with sleep problems, participation in the study will not expose patients to any additional risks. Close monitoring of all possible adverse events related to study medication will take place, and in case any adverse events occur, appropriate measures will be taken in consultation with the attending physician. The Clinical Research Unit (CRU) of the AMC will conduct the monitoring of this trial in compliance with the Medical Research Involving Human Subjects Act (WMO) and ICH-GCP regulations[35]. Annual reports of the study progress will be sent to the Medical Research Ethics

For peer review only

Committee of the Academic Medical Centre Amsterdam. As this trial is considered as a low risk study with a low risk on serious adverse events, no Data and Safety Monitoring Board (DSMB) will be installed or interim analysis performed.

For peer review only

Measurement	Description and instrument	Timepoints baseline	daily	discharge
**Sleep quality**	*Leeds Sleep Evaluation Questionnaire (LSEQ)*[39,43], ten item questionnaire, self-rated, on 0-100 millimetre(mm) line. Validated tool for measuring changes in sleep on four domains; getting to sleep, quality of sleep, ease and awakening from sleep and alertness and behaviour following wakefulness. The Dutch version of the LSEQ is forwards-and backwards translated according to the translation guidelines[44].	x	x	
	Numeric rating scale for overall sleep, an overall numeric rating of quality of sleep. With '0' representing a very bad night's sleep and '10' representing a very good night's sleep.	x	x	
**Sleep pattern**	*The core consensus sleep diary*[45], a ten item sleep log to gain insight in the sleep pattern of a participant.	x	x	
**Factor influencing sleep**	Based on literature[15,46–49], an overview is made of ten influencing factors on sleep in hospitals. Participants can indicate whether these factors influenced their sleep and if so, to what extent.	x	x	
**Objective sleep parameters**	*Actigraphy, MotionWatch 8,* a wristwatch-like device, worn on the non-dominant hand. The actiwatch registers motion in epochs of 30 seconds. Based on motion sleep latency, sleep efficiency, number and duration of wake bouts, time awake after sleep onset in minutes can be calculated.	x	x	
**Cognition**	*Mini-Mental state Examination*[50], A ten item screening tool to evaluate global cognitive function.	x		
	Stroop Colour-Word test[51,52], consists of three subtasks. Used to measure attention, processing speed, cognitive flexibility and working memory.	x	x[1]	
	The Trail Making Test (TMT) A&B [53,54], consists of 2 subtasks, is used to measure visual search, attention, divided attention, processing speed and cognitive flexibility.	x	x[1]	
**Assessment of delirium**	*4 AT,* [55] A four item screening tool to detect attention deficits due to delirium.	x	x[2]	
	DSM-V,[56] the Diagnostic and statistical Manual of the American Psychiatric Association (DSM-5) the most widely used nomenclature by clinicians and researchers for the classification of delirium.	x[3]	x[3]	
**Severity of sleep problems**	*Insomnia severity index*[57], a seven item questionnaire, self-rated, to quantify perceived insomnia severity.	x		
**Depression and anxiety**	*Hospital Anxiety Depression scale (HADS)*[58], a 14-item questionnaire, self-rated, consisting of two seven item subscales looking at depression and anxiety.	x		
**Level of independence**	*Katz-15 ADL index scale,*[59,60] the modified Katz ADL index 15 item-scale, answered with 'yes' or 'no' to evaluate the level of independence.	x		
**Health status**	*Rotterdam symptom check-list (RSCL)*[61], a 30 item questionnaire, self-rated, to assess physical and psychological symptom.	x	x[4]	
	Charlson comorbidity index (CCI),[62] a classification method for comorbid conditions. It is a valid method to estimate risk of death comorbid disease in one year.			x
**Vulnerability (e.g. fall risk)**	Safety management system patient screening *(VMS)*[63]	x		
**Adverse events**	Confusion, delirium, reduced consciousness, falls, aspiration, other additional adverse events (in complication register) and length of hospital stay in days.		x	

Table 1 Overview of the timing of measurements and outcome measures

1. On day three Stroop Colour-Wordtest and TMT are repeated. 2. Only administered if a delirium is suspected. 3. When score on 4AT deviates, detailed assessment of mental status with DSM-V will be done. 4. On day four RSCL is repeated.

DISCUSSION

Although sleeping problems are a common complaint in hospitalised older patients, there is still no evidence based pharmacological treatment available. Most studies on (non-)pharmacological treatment of sleeping problems in older patients were performed in community dwelling older people. However, sleep quality in hospitalised elderly is influenced by other factors than community dwelling older people and might require other therapeutic regimes[64]. For example, light and noise in the room influences the quality of sleep in hospitalised older patients and it is difficult to measure, subsequently standardize for these confounders on the ward. In order to minimize the effect of these potential confounders, we stratified randomisation, in which patients are randomised by hospitals and departments. In this way, the known and unknown confounders are randomised and equally distributed among the groups.

In this study it was not possible to measure sleep quality and quantity using polysomnography, which is the gold standard to measure sleep quality and quantity. Instead of polysomnography, we measured objective sleep parameters indirectly by using an actigraphy, in addition to the self-reported sleep outcome measures.

The MATCH study is the first large randomised controlled trial on the effects of melatonin, temazepam or placebo in hospitalised older patients, which aimed to establish an evidence based pharmacological treatment for sleeping problems in older hospitalised patients. Irrespective of the results of this trial, the outcomes can be used for developing clinical guidelines for the treatment of sleep problems among hospitalized older patients.

ETHICS AND DISSEMINATION

This study was approved by the Medical Research Ethics Committee of the Academic Medical Centre Amsterdam in June 2017. Reference number: 2015_302.The executive boards of the other participating centres have provided local feasibility approval. The trial will be conducted in accordance with the Declaration of Helsinki 1996 and principles of good clinical practice. Any substantial protocol amendments will be submitted to the ethics committee. A register of protocol amendments will be made available in the study protocol.

The grant was an unrestricted grant from the University of Amsterdam Foundation.

Trial methods and results will be reported according to the CONSORT 2010 guidelines[65]. The study findings will be published in relevant peer-reviewed journals.

Trial status

The trial has started on 29th of January 2018.

For peer review only - http://bmjopen.bmj.com/site/about/guidelines.xhtml

Information on trial and research group

Acknowledgements: The authors thank Carolien Jansen (data manager) and the senior citizens of Denktank 60+ Noord for their time, critical appraisal and advise.

Authors contributions: FS and SB contributed equally to this paper. SR is responsible for the original conception of the study and obtaining the grant. FS, SB, SJB, NS, BvM, NV, LH, SR attributed to the design of the study. FS and SB wrote the first draft of the manuscript, all authors contributed to further drafts. All authors read and approved the final draft.

Funding: The MATCH-trial is unrestrictedly financed by the Dalderup foundation. Competing Interests: The authors declare no competing interests.

For peer review only

REFERENCES

1 Enomoto M, Tsutsui T, Higashino S, *et al.* Sleep-related problems and use of hypnotics in inpatients of acute hospital wards. *Gen Hosp Psychiatry* 2010;**32**:276–83. doi:10.1016/j.genhosppsych.2010.01.015

2 Isaia G, Corsinovi L, Bo M, *et al.* Insomnia among hospitalized elderly patients: prevalence, clinical characteristics and risk factors. *Arch Gerontol Geriatr* 2011;**52**:133–7. doi:10.1016/j.archger.2010.03.001

3 Meissner HH, Riemer A, Santiago SM, *et al.* Failure of physician documentation of sleep complaints in hospitalized patients. *West J Med* 1998;**169**:146–9.http://www.pubmedcentral.nih.gov/articlerender.fcgi?artid=1305196&tool=pmcentrez&rendertype=abstract (accessed 17 Jan 2017).

4 Irwin MR, McClintick J, Costlow C, *et al.* Partial night sleep deprivation reduces natural killer and cellular immune responses in humans. *FASEB J* 1996;**10**:643–53.

5 Weinhouse GL. When Perception May Not Meet Reality : Sleep and. Crit. Care Med. 2014;**43**:248–9. doi:10.1097/CCM.0000000000000678

6 Inouye SK, Westendorp RGJ, Saczynski JS. Delirium in elderly people. *Lancet* 2014;**383**:911–22. doi:10.1016/S0140-6736(13)60688-1

7 Watson PL, Ceriana P, Fanfulla F. Delirium: is sleep important? *Best Pract Res Clin Anaesthesiol* 2012;**26**:355–66. doi:10.1016/j.bpa.2012.08.005

8 Chen S, Shi LG, Liang F, *et al.* Exogenous Melatonin for Delirium Prevention: a Meta-analysis of Randomized Controlled Trials. *Mol Neurobiol* 2016;**53**:4046–53. doi:10.1007/s12035-015-9350-8

9 Wu L, Sun D. Sleep duration and falls: a systemic review and meta-analysis of observational studies. *J Sleep Res* 2017;**26**:293–301. doi:10.1111/jsr.12505

10 Leproult R, Copinschi G, Buxton O, *et al.* Sleep loss results in an elevation of cortisol levels the next evening. *Sleep* 1997;**20**:865–70.http://www.ncbi.nlm.nih.gov/pubmed/9415946 (accessed 11 Aug 2017).

11 Kamdar BB, Needham DM, Collop NA. Sleep deprivation in critical illness: its role in physical and psychological recovery. *J Intensive Care Med* 2012;**27**:97–111. doi:10.1177/0885066610394322

12 Nederlande Internisten vereniging. Evidence-based richtlijn nieuwe of verergerende slaapproblemen bij acuut opgenomen ouderen in het ziekenhuis of verpleeghuis. Richtlijn. 2015;:1-89. https://internisten.nl/sites/internisten.nl/files/uploads/Me/i7/Mei7UYMgdwrpvUW8cZWRgQ

For peer review only

/Richtlijn-NIV-Slaapproblemen---Geautoriseerde-versie.pdf (accessed 4 Nov 2017).

13 Young JS, Bourgeois JA, Hilty DM, *et al.* Sleep in hospitalized medical patients, Part 2: Behavioral and pharmacological management of sleep disturbances. *J Hosp Med* 2009;**4**:50–9. doi:10.1002/jhm.397

14 Lenhart SE, Buysse DJ. Treatment of insomnia in hospitalized patients. *Ann Pharmacother* 2001;**35**:1449–57. doi:10.1345/aph.1A040

15 Tamrat R, Huynh-Le M-P, Goyal M. Non-Pharmacologic Interventions to Improve the Sleep of Hospitalized Patients: A Systematic Review. *J Gen Intern Med* 2014;**29**:788. doi:10.1007/S11606-013-2640-9

16 Kanji S, Mera A, Hutton B, *et al.* Pharmacological interventions to improve sleep in hospitalised adults: a systematic review. *BMJ Open* 2016;**6**:e012108. doi:10.1136/bmjopen-2016-012108

17 Frighetto L, Marra C, Bandali S, *et al.* An assessment of quality of sleep and the use of drugs with sedating properties in hospitalized adult patients. *Health Qual Life Outcomes* 2004;**2**:17. doi:10.1186/1477-7525-2-17

18 Gillis CM, Poyant JO, Degrado JR, *et al.* Inpatient pharmacological sleep aid utilization is common at a tertiary medical center. J. Hosp. Med. 2014;**9**:652–7. doi:10.1002/jhm.2246

19 Glass. Sedative hypnotics in older people with insomnia:meta-analysis of risk and benefits. *BMJ* 2005.

20 Cumming RG, Le Couteur DG. Benzodiazepines and risk of hip fractures in older people: a review of the evidence. *CNS Drugs* 2003;**17**:825–37.http://www.ncbi.nlm.nih.gov/pubmed/12921493 (accessed 18 Jan 2017).

21 Brandt J, Leong C. Benzodiazepines and Z-Drugs: An Updated Review of Major Adverse Outcomes Reported on in Epidemiologic Research. *Drugs R D* Published Online First: 1 September 2017. doi:10.1007/s40268-017-0207-7

22 Halfens R, Cox K, Kuppen???Van Merwijk A. Effect of the use of sleep medication in Dutch hospitals on the use of sleep medication at home. J. Adv. Nurs. 1994;**19**:66–70. doi:10.1111/j.1365-2648.1994.tb01052.x

23 Buscemi N, Vandermeer B, Hooton N, *et al.* The efficacy and safety of exogenous melatonin for primary sleep disorders. A meta-analysis. *J Gen Intern Med* 2005;**20**:1151–8. doi:10.1111/j.1525-1497.2005.0243.x

24 de Jonghe A, Korevaar JC, van Munster BC, *et al.* Effectiveness of melatonin treatment on circadian rhythm disturbances in dementia. Are there implications for delirium? A systematic review. *Int J Geriatr Psychiatry* 2010;**25**:1201–8. doi:10.1002/gps.2454

25 Chakraborti D, Tampi DJ, Tampi RR. Melatonin and Melatonin Agonist for Delirium in the Elderly Patients. *Am J Alzheimer's Dis Other Dementiasr* 2015;**30**:119–29.

For peer review only

doi:10.1177/1533317514539379

26 Chen S, Shi L, Liang F, *et al.* Exogenous Melatonin for Delirium Prevention: a Meta-analysis of Randomized Controlled Trials. *Mol Neurobiol* 2016;**53**:4046–53. doi:10.1007/s12035-015-9350-8

27 Brzezinski A. Melatonin in humans.pdf. *N Engl J Med* 1997;**336**:186–95. doi:10.1056/NEJM199701163360306

28 Wade AG, Ford I, Crawford G, *et al.* Efficacy of prolonged release melatonin in insomnia patients aged 55-80 years: quality of sleep and next-day alertness outcomes. *Curr Med Res Opin* 2007;**23**:2597–605. doi:10.1185/030079907X233098

29 Lemoine P, Nir T, Laudon M, *et al.* Prolonged-release melatonin improves sleep quality and morning alertness in insomnia patients aged 55 years and older and has no withdrawal effects. *J Sleep Res* 2007;**16**:372–80. doi:10.1111/j.1365-2869.2007.00613.x

30 Bourne RS, Mills GH, Minelli C. Melatonin therapy to improve nocturnal sleep in critically ill patients: encouraging results from a small randomised controlled trial. *Crit Care* 2008;**12**:R52. doi:10.1186/cc6871

31 Shilo L, Dagan Y, Smorjik Y, *et al.* Effect of melatonin on sleep quality of COPD intensive care patients: a pilot study. *Chronobiol Int* 2000;**17**:71–6.http://www.ncbi.nlm.nih.gov/pubmed/10672435 (accessed 18 Jan 2017).

32 Ibrahim MG, Bellomo R, Hart GK, *et al.* A double-blind placebo-controlled randomised pilot study of nocturnal melatonin in tracheostomised patients. *Crit Care Resusc* 2006;**8**:187–91.http://www.ncbi.nlm.nih.gov/pubmed/16930101 (accessed 18 Jan 2017).

33 Andrade C, Srihari BS, Reddy KP, *et al.* Melatonin in medically ill patients with insomnia: a double-blind, placebo-controlled study. *J Clin Psychiatry* 2001;**62**:41–5.http://www.ncbi.nlm.nih.gov/pubmed/11235927 (accessed 18 Jan 2017).

34 Agha RA, Altman DG, Rosin D. The SPIRIT 2013 statement – Defining standard protocol items for trials. *Int J Surg* 2015;**13**:288–91. doi:10.1016/j.ijsu.2014.12.007

35 ICH. Integrated addendum to ICH e6(R1): Guideline for good clinical practice ICH. 2016.

36 Vural EMS, van Munster BC, de Rooij SE. Optimal dosages for melatonin supplementation therapy in older adults: a systematic review of current literature. *Drugs Aging* 2014;**31**:441–51. doi:10.1007/s40266-014-0178-0

37 Zorginstituut Nederland. Temazepam benzodiazepine agonisten | N05CD07. https://www.farmacotherapeutischkompas.nl/bladeren/preparaatteksten/t/temazepam (accessed 4 Nov 2017).

38 Parrott AC, Hindmarch I. The Leeds Sleep Evaluation Questionnaire in psychopharmacological investigations - a review. *Psychopharmacology (Berl)* 1980;**71**:173–

For peer review only

9.http://www.ncbi.nlm.nih.gov/pubmed/6777817 (accessed 19 Jan 2017).

39 Tarrasch R, Laudon M, Zisapel N. Cross-cultural validation of the Leeds sleep evaluation questionnaire (LSEQ) in insomnia patients. *Hum Psychopharmacol* 2003;**18**:603–10. doi:10.1002/hup.534

40 Van Beuningen J, van der Houwen K, Moonen L. Measuring well-being An analysis of different response scales. The Hague/Heerlen: 2014. www.cbs.nl

41 Zisapel N, Nir T. Determination of the minimal clinically significant difference on a patient visual analog sleep quality scale. *J Sleep Res* 2003;**12**:291–8.http://www.ncbi.nlm.nih.gov/pubmed/14633240 (accessed 18 Jan 2017).

42 de Jonghe A, van Munster BC, Goslings JC, *et al.* Effect of melatonin on incidence of delirium among patients with hip fracture: a multicentre, double-blind randomized controlled trial. *CMAJ* 2014;**186**:E547-56. doi:10.1503/cmaj.140495

43 Parrott AC, Hindmarch I. Factor analysis of a sleep evaluation questionnaire. *PsycholMed* 1978;**8**:325–9. doi:10.1017/S0033291700014379

44 Beaton DE, Bombardier C, Guillemin F, *et al.* Guidelines for the process of cross-cultural adaptation of self-report measures. *Spine (Phila Pa 1976)* 2000;**25**:3186–91.http://www.ncbi.nlm.nih.gov/pubmed/11124735 (accessed 20 Aug 2017).

45 Carney CE, Buysse DJ, Ancoli-Israel S, *et al.* The consensus sleep diary: standardizing prospective sleep self-monitoring. *Sleep* 2012;**35**:287–302. doi:10.5665/sleep.1642

46 Park MJ, Kim KH. What affects the subjective sleep quality of hospitalized elderly patients? *Geriatr Gerontol Int* 2016;:n/a-n/a. doi:10.1111/ggi.12743

47 Bano M, Chiaromanni F, Corrias M, *et al.* The Influence of Environmental Factors on Sleep Quality in Hospitalized Medical Patients. *Front Neurol* 2014;**5**:267. doi:10.3389/fneur.2014.00267

48 Ersser S, Wiles A, Taylor H, *et al.* The sleep of older people in hospital and nursing homes. *J Clin Nurs* 1999;**8**:360–8.http://www.ncbi.nlm.nih.gov/pubmed/10624252 (accessed 13 Mar 2017).

49 Dobing S, Frolova N, McAlister F, *et al.* Sleep quality and factors influencing self-reported sleep duration and quality in the general internal medicine inpatient population. *PLoS One* 2016;**11**:1–6. doi:10.1371/journal.pone.0156735

50 Folstein MF, Folstein SE, McHugh PR. 'Mini-mental state'. A practical method for grading the cognitive state of patients for the clinician. *J Psychiatr Res* 1975;**12**:189–98.http://www.ncbi.nlm.nih.gov/pubmed/1202204 (accessed 21 Sep 2016).

51 Stroop JR. Studies of interference in serial verbal reactions. *J Exp Psychol* 1935;**XVIII**:643–62.

52 Hammes J. De stroop Kleur-woord test: handleiding [The Stroop Color-word test: manual]. Lisse: 1997.

53 Army Individual Test Battery. Manual of Directions and Scoring. *Washington, DC War Dep Adjut*

For peer review only

Gen Off 1944.

54 M.D. Lezak DBH. *Neuropsychological assessment*. 4th ed. New York: : Oxford University Press 2004.

55 Bellelli G, Morandi A, Davis DHJ, *et al.* Validation of the 4AT, a new instrument for rapid delirium screening: a study in 234 hospitalised older people. *Age Ageing* 2014;**43**:496–502. doi:10.1093/ageing/afu021

56 American Psychiatric Association. *Diagnostic and statistical manual of mental disorders*. 5th ed. Washington, DC: 2013.

57 Bastien CH, Vallières A, Morin CM. Validation of the Insomnia Severity Index as an outcome measure for insomnia research. *Sleep Med* 2001;**2**:297–307.http://www.ncbi.nlm.nih.gov/pubmed/11438246 (accessed 11 Aug 2017).

58 Cosco TD, Doyle F, Ward M, *et al.* Latent structure of the Hospital Anxiety And Depression Scale: a 10-year systematic review. *J Psychosom Res* 2012;**72**:180–4. doi:10.1016/j.jpsychores.2011.06.008

59 Laan W, Zuithoff NPA, Drubbel I, *et al.* Validity and reliability of the Katz-15 scale to measure unfavorable health outcomes in community-dwelling older people. *J Nutr Health Aging* 2014;**18**:848–54. doi:10.1007/s12603-014-0479-3

60 Weinberger M, Samsa GP, Schmader K, *et al.* Comparing proxy and patients' perceptions of patients' functional status: results from an outpatient geriatric clinic. *J Am Geriatr Soc* 1992;**40**:585–8.http://www.ncbi.nlm.nih.gov/pubmed/1587975 (accessed 19 Jan 2017).

61 de Haes JC, van Knippenberg FC, Neijt JP. Measuring psychological and physical distress in cancer patients: structure and application of the Rotterdam Symptom Checklist. *Br J Cancer* 1990;**62**:1034–8. doi:10.1038/bjc.1990.434

62 Charlson ME, Pompei P, Ales KL, *et al.* A new method of classifying prognostic comorbidity in longitudinal studies: development and validation. *J Chronic Dis* 1987;**40**:373–83.http://www.ncbi.nlm.nih.gov/pubmed/3558716 (accessed 25 Oct 2016).

63 Oud FMM, de Rooij SEJA, Schuurman T, *et al.* [Predictive value of the VMS theme 'Frail elderly': delirium, falling and mortality in elderly hospital patients]. *Ned Tijdschr Geneeskd* 2015;**159**:A8491.http://www.ncbi.nlm.nih.gov/pubmed/25761294 (accessed 4 Nov 2017).

64 Wesselius HM, Van Den Ende ES, Alsma J, *et al.* Quality and quantity of sleep and factors associated with sleep disturbance in hospitalized patients. *JAMA Intern Med* 2018;**178**:1165–71. doi:10.1001/jamainternmed.2018.2669

65 Moher D, Hopewell S, Schulz KF, *et al.* CONSORT 2010 explanation and elaboration: Updated guidelines for reporting parallel group randomised trials. *Int J Surg* 2012;**10**:28–55. doi:10.1016/j.ijsu.2011.10.001

For peer review only

Figure 1. Flow chart of trial design summary

For peer review only

For peer review only - http://bmjopen.bmj.com/site/about/guidelines.xhtml

Figure 1 Summary of MATCH trial

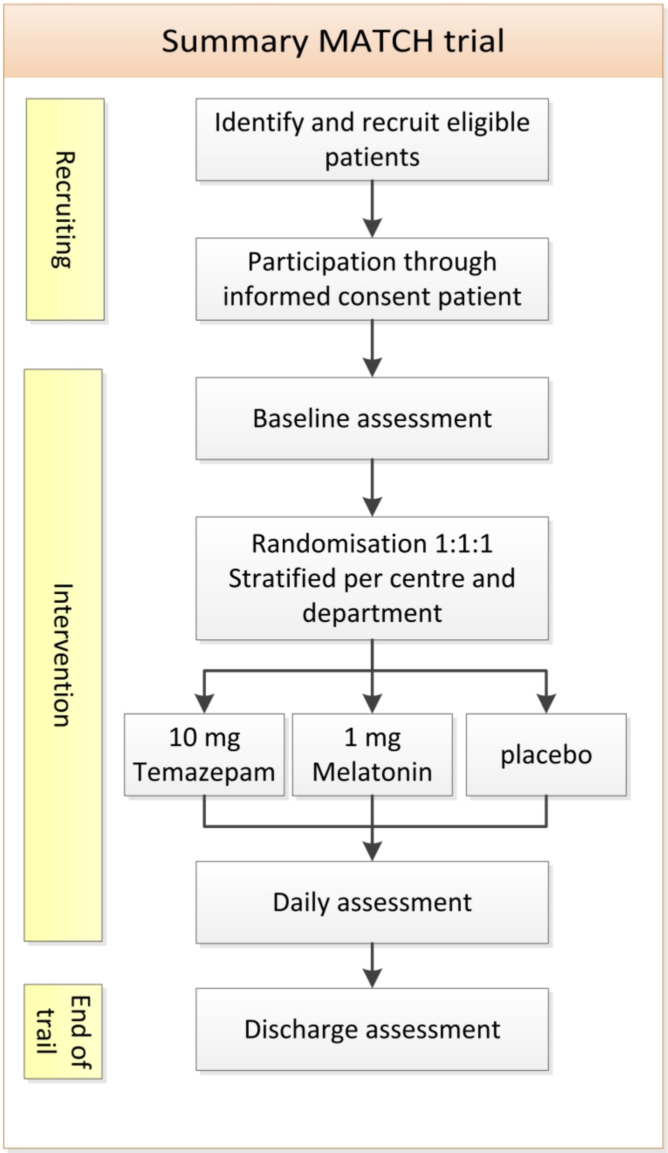

Figure 1. Flow chart of trial design summary

Reporting checklist for protocol of a clinical trial.

Based on the SPIRIT guidelines.

Instructions to authors

Complete this checklist by entering the page numbers from your manuscript where readers will find each of the items listed below.

Your article may not currently address all the items on the checklist. Please modify your text to include the missing information. If you are certain that an item does not apply, please write "n/a" and provide a short explanation.

Upload your completed checklist as an extra file when you submit to a journal.

In your methods section, say that you used the SPIRIT reporting guidelines, and cite them as:

Chan A-W, Tetzlaff JM, Altman DG, Laupacis A, Gøtzsche PC, Krleža-JeriĆ K, Hróbjartsson A, Mann H, Dickersin K, Berlin J, Doré C, Parulekar W, Summerskill W, Groves T, Schulz K, Sox H, Rockhold FW, Rennie D, Moher D. SPIRIT 2013 Statement: Defining standard protocol items for clinical trials. Ann Intern Med. 2013;158(3):200-207

For peer review only

		Reporting Item	Page Number
Title	#1	Descriptive title identifying the study design, population, interventions, and, if applicable, trial acronym	1
Trial registration	#2a	Trial identifier and registry name. If not yet registered, name of intended registry	2
Trial registration: data set	#2b	All items from the World Health Organization Trial Registration Data Set	See note 1
Protocol version	#3	Date and version identifier	1
Funding	#4	Sources and types of financial, material, and other support	12
Roles and responsibilities: contributorship	#5a	Names, affiliations, and roles of protocol contributors	1,12
Roles and responsibilities: sponsor contact information	#5b	Name and contact information for the trial sponsor	See note 2
Roles and	#5c	Role of study sponsor and funders, if any, in study design; collection, management,	See note 3

responsibilities: sponsor and funder		analysis, and interpretation of data; writing of the report; and the decision to submit the report for publication, including whether they will have ultimate authority over any of these activities	
Roles and responsibilities: committees	#5d	Composition, roles, and responsibilities of the coordinating centre, steering committee, endpoint adjudication committee, data management team, and other individuals or groups overseeing the trial, if applicable (see Item 21a for data monitoring committee)	1,4
Background and rationale	#6a	Description of research question and justification for undertaking the trial, including summary of relevant studies (published and unpublished) examining benefits and harms for each intervention	3
Background and rationale: choice of comparators	#6b	Explanation for choice of comparators	3
Objectives	#7	Specific objectives or hypotheses	4
Trial design	#8	Description of trial design including type of trial (eg, parallel group, crossover, factorial, single group), allocation ratio, and framework (eg, superiority, equivalence, non-inferiority, exploratory)	4
Study setting	#9	Description of study settings (eg, community clinic, academic hospital) and list of countries where data will be collected. Reference to where list of study sites can be obtained	4
Eligibility criteria	#10	Inclusion and exclusion criteria for participants. If applicable, eligibility criteria for study centres and individuals who will perform the interventions (eg, surgeons, psychotherapists)	5
Interventions: description	#11a	Interventions for each group with sufficient detail to allow replication, including how and when they will be administered	6,7
Interventions: modifications	#11b	Criteria for discontinuing or modifying allocated interventions for a given trial participant (eg, drug dose change in response to harms, participant request, or improving / worsening disease)	7
Interventions: adherance	#11c	Strategies to improve adherence to intervention protocols, and any procedures for monitoring adherence (eg, drug tablet return; laboratory tests)	See note 4
Interventions: concomitant care	#11d	Relevant concomitant care and interventions that are permitted or prohibited during the trial	6,7
Outcomes	#12	Primary, secondary, and other outcomes, including the specific measurement variable	7,8,9

For peer review only

		(eg, systolic blood pressure), analysis metric (eg, change from baseline, final value, time to event), method of aggregation (eg, median, proportion), and time point for each outcome. Explanation of the clinical relevance of chosen efficacy and harm outcomes is strongly recommended	
Participant timeline	#13	Time schedule of enrolment, interventions (including any run-ins and washouts), assessments, and visits for participants. A schematic diagram is highly recommended (see Figure)	7 and figure 1
Sample size	#14	Estimated number of participants needed to achieve study objectives and how it was determined, including clinical and statistical assumptions supporting any sample size calculations	8
Recruitment	#15	Strategies for achieving adequate participant enrolment to reach target sample size	5
Allocation: sequence generation	#16a	Method of generating the allocation sequence (eg, computer-generated random numbers), and list of any factors for stratification. To reduce predictability of a random sequence, details of any planned restriction (eg, blocking) should be provided in a separate document that is unavailable to those who enrol participants or assign interventions	6
Allocation concealment mechanism	#16b	Mechanism of implementing the allocation sequence (eg, central telephone; sequentially numbered, opaque, sealed envelopes), describing any steps to conceal the sequence until interventions are assigned	6
Allocation: implementation	#16c	Who will generate the allocation sequence, who will enrol participants, and who will assign participants to interventions	6
Blinding (masking)	#17a	Who will be blinded after assignment to interventions (eg, trial participants, care providers, outcome assessors, data analysts), and how	6
Blinding (masking): emergency unblinding	#17b	If blinded, circumstances under which unblinding is permissible, and procedure for revealing a participant's allocated intervention during the trial	6
Data collection plan	#18a	Plans for assessment and collection of outcome, baseline, and other trial data, including any related processes to promote data quality (eg, duplicate measurements, training of assessors) and a description of study instruments (eg, questionnaires, laboratory tests) along with their reliability and validity, if known. Reference to where data collection forms can be found, if not in the protocol	7,8,9,10
Data collection plan: retention	#18b	Plans to promote participant retention and complete follow-up, including list of any outcome data to be collected for participants who discontinue or deviate from intervention protocols	See note 5

For peer review only

Data management	#19	Plans for data entry, coding, security, and storage, including any related processes to promote data quality (eg, double data entry; range checks for data values). Reference to where details of data management procedures can be found, if not in the protocol	9
Statistics: outcomes	#20a	Statistical methods for analysing primary and secondary outcomes. Reference to where other details of the statistical analysis plan can be found, if not in the protocol	8,9
Statistics: additional analyses	#20b	Methods for any additional analyses (eg, subgroup and adjusted analyses)	8,9
Statistics: analysis population and missing data	#20c	Definition of analysis population relating to protocol non-adherence (eg, as randomised analysis), and any statistical methods to handle missing data (eg, multiple imputation)	See note 6
Data monitoring: formal committee	#21a	Composition of data monitoring committee (DMC); summary of its role and reporting structure; statement of whether it is independent from the sponsor and competing interests; and reference to where further details about its charter can be found, if not in the protocol. Alternatively, an explanation of why a DMC is not needed	9
Data monitoring: interim analysis	#21b	Description of any interim analyses and stopping guidelines, including who will have access to these interim results and make the final decision to terminate the trial	9
Harms	#22	Plans for collecting, assessing, reporting, and managing solicited and spontaneously reported adverse events and other unintended effects of trial interventions or trial conduct	8,9
Auditing	#23	Frequency and procedures for auditing trial conduct, if any, and whether the process will be independent from investigators and the sponsor	n/a
Research ethics approval	#24	Plans for seeking research ethics committee / institutional review board (REC / IRB) approval	11
Protocol amendments	#25	Plans for communicating important protocol modifications (eg, changes to eligibility criteria, outcomes, analyses) to relevant parties (eg, investigators, REC / IRBs, trial participants, trial registries, journals, regulators)	11
Consent or assent	#26a	Who will obtain informed consent or assent from potential trial participants or authorised surrogates, and how (see Item 32)	5
Consent or assent: ancillary studies	#26b	Additional consent provisions for collection and use of participant data and biological specimens in ancillary studies, if applicable	n/a
Confidentiality	#27	How personal information about potential and enrolled participants will be collected, shared, and maintained in order to protect confidentiality before, during, and after the	9

For peer review only

		trial	
Declaration of interests	#28	Financial and other competing interests for principal investigators for the overall trial and each study site	11
Data access	#29	Statement of who will have access to the final trial dataset, and disclosure of contractual agreements that limit such access for investigators	n/a, see note 3
Ancillary and post trial care	#30	Provisions, if any, for ancillary and post-trial care, and for compensation to those who suffer harm from trial participation	7
Dissemination policy: trial results	#31a	Plans for investigators and sponsor to communicate trial results to participants, healthcare professionals, the public, and other relevant groups (eg, via publication, reporting in results databases, or other data sharing arrangements), including any publication restrictions	2,11
Dissemination policy: authorship	#31b	Authorship eligibility guidelines and any intended use of professional writers	11
Dissemination policy: reproducible research	#31c	Plans, if any, for granting public access to the full protocol, participant-level dataset, and statistical code	n/a
Informed consent materials	#32	Model consent form and other related documentation given to participants and authorised surrogates	See note 8
Biological specimens	#33	Plans for collection, laboratory evaluation, and storage of biological specimens for genetic or molecular analysis in the current trial and for future use in ancillary studies, if applicable	n/a

Author notes

1. n/a see NTR registration. URL: http://www.trialregister.nl/trialreg/admin/rctview.asp?TC=6908
2. Universiteitsfonds UvA, contact information principal investigator (Professor Sophia E.de Rooij) page 1
3. n/a Investigator initiated, unrestricted (see page 10)
4. Patients are not admitted to the hospital for sleeping problems, maximum duration of the study is 10 days. Although patients will often be discharged from the hospital before that.
5. n/a maximum of 10 study days (see also note 4)
6. 7 missing data n/a for this statistical method
7. n/a no interim analysis

For peer review only

8. n/a consent form approved by medical ethic commitee, the forms are in dutch and therefore not added

The SPIRIT checklist is distributed under the terms of the Creative Commons Attribution License CC-BY-ND 3.0. This checklist was completed on 29. June 2018 using https://www.goodreports.org/, a tool made by the EQUATOR Network in collaboration with Penelope.ai

For peer review only
